# Neighboring Groups and Political Attacks

**DOI:** 10.1177/10659129251365875

**Published:** 2025-08-27

**Authors:** Randy Besco, Sergio Garcia-Rios, Julius Lagodny, Nazita Lajevardi, Kassra AR Oskooii, Erin Tolley

**Affiliations:** 1 7938 University of Toronto, Toronto, ON, Canada; 2 12330 University of Texas, Austin, TX, USA; 3 38962 Hertie School, Berlin, Germany; 4 3078 Michigan State University, East Lansing, MI, USA; 5 5972 University of Delaware, Newark, DE, USA; 6 6339 Carleton University, Ottawa, ON, Canada

**Keywords:** elections and voting, racism, discrimination, immigration, Latino politics, Asian politics, Canada, American politics

## Abstract

Explicit racism in political campaigns is rising, with politicians openly disparaging immigrant, racial, and religious minorities. Group membership plays a central role in politics, and people often respond more strongly to attacks on their own group than others. What if the attack is directed toward a group that an individual does *not* belong to, but with which they have a logical, social, or psychological connection? We call these “neighboring” groups. We use survey experiments with immigrant and non-immigrant Latino Americans and South Asian Canadians to understand the effect of exposure to campaign videos that disparage immigrants or Latinos/South Asians. Members of neighboring groups report emotions and candidate evaluations that are very similar to those of directly targeted groups. These findings point to the importance of neighboring groups and suggest that social and psychological connections can produce effects as large as actual group membership.

## Introduction

Explicit racially derogatory appeals are increasingly common in political speech (Stephens-Dougan, 2021; Valentino et al., 2002). In American politics, President Trump rose to prominence on the racist birther myth (Jardina and Traugott, 2019), a proposal to a build wall to keep out Mexicans and other immigrants (Wallace and Zepeda-Millán, 2020), and to ban Muslims from the country (Oskooii et al., 2021). However, racial derogation by political elites is not unique to the US context. In fact, politicians from far-right parties in many countries have openly and routinely disparaged immigrants and minorities (Gagnon and Larios, 2021; Noury and Roland, 2020). In Austria, politicians have compared migrants to rats (Stone, 2019), in Germany to a compost heap (Chambers, 2016), and in Denmark said that they should be shot at (Boffey, 2016). In Canada, the People’s Party installed campaign billboards with the slogan “say no to mass immigration,” (Smith, 2019) and proposed a values test to screen out Muslims with “barbarian ideology” (Salutin, 2024).

Members of the groups generally respond to prejudicial remarks by punishing the speaker (e.g., Bonilla et al., 2022). This response is consistent with a wide range of research demonstrating that membership and identification with a social group affect people’s attitudes and behavior. Work on partisanship, race, and nationalism argues that identities affect perceptions of the economy (Gerber and Huber, 2010), responses to corruption (Solaz et al., 2019), turnout (Rau, 2022), and many other factors. In particular, attacks and criticisms of one’s group often lead to strong emotional reactions, backlash, and political mobilization (Garcia-Rios et al., 2018; Pérez, 2015).

Less is known about how members of “neighboring” groups respond to racist political speech. Neighboring groups are those comprised of people who are not members of the targeted group but who do belong to communities with logical, social, or psychological links. Neighboring groups could include partisans of coalition partners, nationalities from the same region, or native-born members of primarily immigrant ethnic communities. Are the effects of political attacks the same for targeted and neighboring groups?

On the one hand, there are reasons to expect the effects of racially derogatory appeals to present primarily for ingroup members, rather than neighboring groups. Standard social identity theory and policy threat theory explains reactions of in-group members but have no clear implications for non-group members. More specifically, research on immigration and ethnicity further suggests neighboring group effects will be decidedly smaller than ingroup effects (Garcia-Rios et al., 2018; Pantoja and Segura, 2003). In recent years, a minority of Black and Latino voters have shifted rightwards in a “racial realignment” despite explicitly racist positions by Trump and other Republicans, perhaps due to their racial attitudes, or as a way to signal their distinctiveness from immigrants (Cuevas-Molina, 2023; Fraga et al., 2025; Hickel Jr et al., 2024).

On the other hand, more recent work on “proximal contact” of family or community suggests threat may apply more broadly (Walker, 2020). Other research on “people of color” identity suggests the development of superordinate identity (Pérez, 2021), which can lead to solidarity (Kim et al., 2025) between groups like Latino and Asian Americans.

The concept of neighboring groups is useful in extending these prior theories in three ways. First, these are groups which the individual does *not* say they are a member of, which complicates standard social identity ingroup accounts. Second, policy threat literature usually points to policies that threaten the target, but here we focus on circumstances without a direct group threat to a person’s ingroup. Instead, effects on non-targeted people emphasize the extent to which policy threat can be about *beliefs* about threats that are implied, possible in the future, or to family and community, linking together several strands of research. Finally, we suggest neighboring identities is broader than just race, and it could apply to other categories like nationality, political party, and other social identities. Although we do not test the above mechanisms here, these extensions can show how existing theories can also help explain effects on non-group members.

Our objective here is to evaluate the presence (or absence) of neighboring group effects in response to explicit political derogation, and to compare their strength relative to effects on targeted group members. We test this with two survey experiments conducted in the United States with a Latino sample^1^ and in Canada with a South Asian sample. As discussed in the case selection section below, these are the two largest immigrant-based ethnic groups in their respective countries but otherwise have substantial variation, with major differences in political salience (Konitzer et al., 2019; Ramirez and Peterson, 2020), political participation (Fraga, 2018; Soroka et al., 2008), the proportion of immigrants (Statistics Canada, 2022, U.S. Census Bureau, 2022), and other factors. Testing our hypothesis across two ethnically distinct populations in different national contexts strengthens confidence that any observed dynamics are not confined to a single social, political, or cultural setting.

The treatment is a social media style fictional campaign ad, combining photos, text, music, and voice-over actors. The video draws on content from real advertisements, speeches, and statements by politicians. There are three treatment arms, where the politician (a) attacks immigrants (without specific ethnic or racial groups identified), (b) attacks a specific panethnic group (Latinos in the U.S. study and South Asians in the Canadian study), or (c) talks about jobs and economy (control). The samples include both immigrants and non-immigrants, so these three treatments allow within and between group comparisons to distinguish the effects of being a member of the target group, or a member of the neighboring group. Namely, we evaluate whether native-born individuals will react differently to negative campaigning about foreign-born individuals compared to negative campaigning about their own panethnic group.

In line with previous work, we find large effects for members of the targeted groups exposed to ads targeting their ingroup, both in terms of candidate evaluations and emotional reactions. Moreover, in both studies, we find that respondents react similarly to derogatory messaging whether they are the specific target of the attack or simply a member of a neighboring group. These findings demonstrate that neighboring identities can produce effects that are quite large and substantively similar to the targeted group identity.

This research makes several contributions. First, it demonstrates that political messaging targeted at one group can significantly influence individuals in neighboring identity groups, challenging the conventional focus on direct group-target effects. In fact, we find that the size of these neighboring group effects is very similar to those of the targeted group effects. Second, by examining Latinos in the U.S. and South Asians in Canada, the findings highlight the cross-national applicability of our neighboring groups theory. Overall, our study demonstrates that racial or ethnic derogation by political elites may inadvertently mobilize broader coalitions, including those beyond the targeted group, potentially limiting the divisive intent of such rhetoric.

## Social Groups and Responses to Derogation

A wide range of research demonstrates that membership in a social group affects people’s attitudes and behaviors. The social psychological perspective on partisanship, for example, holds that identification with a party has a causal effect on many factors such as perceptions of the economy (Okolikj and Hooghe, 2022), public policy (Williams et al., 2022), and views of other parties (Simas et al., 2020), both in the United States and in Canada (Merkley, 2021; Sheffer, 2020).

What are the motivations and scope conditions of the effects of group membership? Most theories emphasize two broad sources of motivation for group membership effects: symbolic concerns tied to status and self-worth, and perceived threats of material or political harm. The former is usually explained by social identity theory as a desire for collective or self-esteem, and a need to defend and maintain group’s status (Cichocka et al., 2024; Martiny and Rubin, 2016). People then respond to threats or denigration of their group by contestation, such as hostility toward the threatening outgroup (Branscombe and Wann, 1994) or collective action (Simon and Klandermans, 2001).

Conversely, policy or political threat literature focuses on how perceived material threats—those affecting a person’s livelihood, rights, or safety—can prompt political action. Much of the research examines how Latinos increase their political participation in response to xenophobia and immigration-related threats (Pantoja and Segura 2003; Reny et al. 2018; White 2016), although note McNeely et al. (2022) find lower rather than higher turnout. There are similar findings of increased engagement for Black Americans (Platt 2008), Muslim Americans (Schoettmer, 2015), Arab Americans (Cho et al., 2006), and Palestinians (Weiss et al., 2023).

While policy threat and social identity research locate motivation in slightly different ways, the implications are the same: as a member of a group, a person responds more strongly to derogation and threats against their group relative to non-members. This is further articulated by research on political discrimination, which integrates insights from social identity and policy threat literature to argue that laws, symbols, or political campaigns targeting groups can mobilize group members to engage in collective action for substantive or expressive purposes (Oskooii 2016, 2020).

What are the scope conditions of this dynamic? Most research presumes that any effects only apply to primary group members, and reactions to group-related stimuli are contingent on the strength or centrality of one’s identity (e.g., Besco, 2015; Ellemers et al., 1997; Huddy et al., 2015; Valenzuela and Michelson, 2016). In fact, it is standard practice to measure group identity only among individuals who self-identify as members. Similarly, the literature on policy threat typically theorizes that group-targeted threats mobilize primary group members, without fully considering whether secondary or neighboring group members might respond similarly.

There are good reasons to expect that the scope of group effects may extend beyond directly targeted populations. Policy threat research is concerned, at least implicitly, with perceived threat, rather than the formal content of a policy. For example, an all male legislature can be perceived as a threat to women’s interests (Clayton et al., 2023), or the Patriot Act as threatening to Arab Americans (Cho et al., 2006), even in the absence of explicit targeting the group. Since policies often have broader social or symbolic consequences than their text implies, individuals may perceive themselves as affected—rightly or wrongly—even when they are not part of the explicitly named group.

Developments in social identity theory have also broadened the scope of what constitutes group effects. Research on superordinate or common ingroup identity theory (e.g., Gaertner et al., 1994; Transue, 2007) demonstrates that when a more inclusive, overarching group identity is made salient, it expands the boundaries of the ingroup, thereby altering who is perceived as part of “us.”

Motivation and scope are also combined in different ways. Group consciousness is identity tied to ideological beliefs, including the group’s social status and whether collective action could improve it (Miller et al. 1981). It is these ideological beliefs that give identity a “political kick,” as Junn (2006) puts it. Similarly, linked fate is a heuristic that suggests that what affects the group also affects one’s self personally (Dawson, 1994) and provides a cognitive bridge between the individual and the group (Donnelly, 2021). Although most of this work is observational and implies that these are relatively stable beliefs, a notable early experiment (Junn and Masuoka, 2008) showed that exposure to a Chinese-American cabinet member increased linked fate and racial political identity of Asian Americans. Recent work by Pérez and colleagues connects the motivation and scope dimensions with identity, arguing that marginalization and discrimination can lead to a superordinate “people of color” identity, encompassing multiple minority groups (Pérez 2021). Furthermore, research on solidarity among people of color suggests behavioral coordination. Asian Americans and Latinos, for example, share marginalization due to perceived foreignness, which leads them to support policies benefiting other communities of color—even when not directly impacted (Kim et al., 2025).

## Neighboring Groups

Building on this literature, we turn our attention to “neighboring” groups, by which we define as people who are not formally members of the group but who belong to similar or related groups. The nature of their connection might be quite diverse but could be based on logical, psychological, or social links. For example, origins in a similar part of the world, such as Ukrainians and Poles, or ideological or strategic connections such as partisans of coalition partners. They could also be connected through familial or interpersonal networks, such as the affinity between first- and second-generation members of immigrant communities. The study presented here is agnostic to which theoretical motivation is at work, partly because we think it is likely there are multiple overlapping motivations. The usefulness of our concept is that it distinguishes between types of outgroups, some of which are quite distant or disconnected, and others are similar and related.

The neighboring groups concept broadens the scope of prior theories by extending it beyond membership in a directly targeted group. Traditional identity theory holds non-members won’t act like members, and the people of color framework (Pérez, 2021) argues that shared racial identity can extend membership. However, superordinate identities can form quickly, as shown by minimal group experiments and Brexit research (Hobolt et al., 2021). We suggest superordinate identities may be more amorphous and easily created than sometimes appreciated. Similarly, political responses often stem from perceived—not formal—policy threats, especially when harm to family or community is involved (Morris and Kelsey, 2024; Walker, 2020), thus applying to non-group members. Expanding our conceptual toolkit to include neighboring groups helps capture these dynamics.

If non-immigrants are taken as the neighboring group and immigrants as the ingroup, one concern is that non-immigrants might identify as immigrants given their social and historical links. If so, neighboring effects might just be standard ingroup effects. This possibility raises subtle questions about the nature of identity: can one identify with a group to which one does not belong? Perhaps, partly a function of conceptual ambiguity resulting from social construction, but also because identification can be quite flexible, encompassing metaphorical and emotional connections. To the extent that non-immigrants identify as immigrants, it illustrates the neighboring groups concept by showing how the boundaries of identification can extend beyond “objective” group membership to include related groups.

Prior research offers some guidance on whether native-born coethnics respond to immigrant-directed threats, which might be considered a neighboring group. Proposition 187, which targeted immigrants, increased political knowledge (Pantoja and Segura, 2003) and perceived discrimination (Gutierrez, 2025) of immigrant, but not native-born, Latinos. Immigration laws increase linked fate of foreign-born Latinos (Vargas Jr et al., 2017), although Maltby et al. (2020) find enforcement effects on native-born but not foreign-born Latinos. Pérez (2015) shows stronger effects of a xenophobic treatment about “illegal” immigration among low-acculturation Latinos, although this combines includes immigration and other variables. There is evidence that being an immigrant is correlated with panethnic group consciousness for Latinos (Masuoka 2006), but not Asian Americans (Nicholson Jr and Mei, 2023). Similarly, Garcia-Rios (2018) find that Trump’s attacks on Mexicans more strongly affected how Mexican Americans evaluated him than all Latinos in general. Though mixed, these results generally imply smaller or nonexistent responses among neighboring groups, in this case native-born coethnics.

Yet, there are theoretical reasons to expect members of neighboring groups to react similarly as primary group members. As discussed above, non-immigrants might identify as an immigrant to a degree, especially if they have family and friends who are immigrants, or if others perceive them as immigrants. In the PoC identity model (Pérez, 2021), this is analogous to identifying with people of color as a group, despite claiming not to be a person of color. However, in this scenario, we would expect the immigrant identification of non-immigrants, on average, to be less central than for people who are in fact foreign-born, and their reactions to derogation to be correspondingly weaker.

Similarly, policy threat might apply to non-group members indirectly. In the U.S., the media often link immigration and Latino people (Abrajano and Hajnal, 2015; Bedolla, 2005; Pérez, 2016), and Latinos may do so themselves. Conversely, they may be aware that others connect immigration with ethnicity, and thus conclude that an anti-immigrant politician is also anti-Latino.

Coethnic non-immigrants might care about the impact of policies on their immigrant family and friends, as “proximal contact” research shows with the effects of family members interacting with the criminal justice system (Walker, 2020), and neighborhoods with people killed by the police (Drolc and Shoub, 2024; Morris and Kelsey, 2024).

Aside from their personal interaction with a policy, people might also have principled beliefs: they might simply believe racism is wrong, and they therefore reject racist policies or candidates (e.g., Mendelberg, 2001; Tesler, 2020; Valentino et al., 2018). This explanation is especially likely for non-white groups; as Bonilla et al. (2022) show, Black Americans are particularly sensitive to outgroup derogation due to their own experience of racism. They therefore object to attacks on Muslims even when they do not personally identify with the group.

In general, prior research presents conflicting expectations. On the one hand, there is evidence that members of a targeted group respond to political threats, while neighboring or non-targeted group members show weaker—or no—responses. On the other hand, there are reasons to expect that “neighboring” groups may react. This may occur due to partial identification (of non-members), perceived threat (even if not directly targeted), social ties to the group, or principled, sympathetic concern. In addition, much of the existing research centers on Latinos in the U.S., leaving it unclear whether the results generalize to other racial and ethnic groups, or other national contexts. By extending the analysis to South Asians outside the U.S. and incorporating the concept of neighboring identities, this study aims to clarify the reach and impact of group-based political derogation.

In sum, we ask: Do individuals respond to attacks on neighboring groups in the same way they respond to attacks on their own group, or are effects more limited? Specifically, do immigrant and non-immigrant Latinos and South Asians respond similarly to anti-immigrant political rhetoric? Drawing on prior research, we offer the following hypotheses:

H1 Immigrant (foreign-born) and non-immigrant (native-born) Latinos/South Asians will react negatively to candidates who attack their respective panethnic groups.

H2 Non-immigrant (native-born) Latinos/South Asians will display stronger negative reactions to candidates who attack their panethnic (Latino/South Asian) group than to candidates who attack immigrants.

H3 Non-immigrant (native-born) Latinos/South Asians will display similar negative reactions as immigrant (foreign-born) Latinos/South Asians to candidates who attack their neighboring group (i.e., immigrants).

To test our hypotheses, we conducted two experimental studies using treatments that vary the explicit target of a political attack. Our treatments include an attack on immigrants generally, as well as an attack on a specific panethnic group. Responses to these treatments are compared to a control condition that does not raise any immigrant or ethnic group identity but instead focuses on the economy. To evaluate these differences, we focus on two sets of outcome variables: emotional reactions and candidate evaluations.

## Cases: Latinos in the U.S. and South Asians in Canada

The research design tests our hypotheses using two different ethnic group samples in two countries: Latinos in the U.S. and South Asians in Canada. This comparative approach helps to mitigate idiosyncratic effects associated with a single group or country, such as political context, media environment, or immigrant origin, while increasing the generalizability and confidence in the results.

Latinos are the largest immigrant ethnic group in the U.S. comprising about 18% of the population, of which 33% of those are foreign-born. Mexico is the largest country of origin (62%), with the rest from Central and South American countries (Pew Research, 2023, U.S. Census Bureau, 2022). There is a long history of Latino immigration to the U.S., with substantial informal migrant flows until reforms in recent decades (Durand et al., 1999). In addition, there are Latino populations with origins in the U.S. due to wars of territorial expansion, including in most of the southwest states. This means that a substantial portion of the Latino population are not immigrants, nor recent descendants of them.

Latinos occupy an important place in American politics, and their salience now arguably rivals that of Black Americans (Ramirez and Peterson 2020). Latinos have long been recognized as a growing demographic group with increasing political influence, even as that influence has lagged behind expectations (De la Garza, 2004; Rouse, 2013). Turnout and political participation are still somewhat lower among Latinos in the U.S. than White/Anglo-Americans (Fraga, 2018), as are political donations (Grumbach and Sahn, 2020).

When they do participate in politics, Latinos tend to cast votes for Democratic candidates (e.g., 61% in 2020 voted for Biden over Trump (Pew Research, 2024). Most Latinos support progressive immigration policies, including allowing undocumented children to stay and seek legal status (Pew Research, 2021). Because Latinos are the primary targets and victims of immigration enforcement, they disproportionately bear the discriminatory impacts of immigration policies in the U.S. (Walker et al. 2020). Many Latino families—including those with native-born members—regularly grapple with how to engage with immigration enforcement (Asad, 2023). Moreover, punitive immigration laws (Vargas Jr et al., 2017) and local immigration enforcement are correlated with stronger Latino linked fate.

However perhaps surprisingly, a non-negligible segment Latinos are somewhat receptive to more conservative and anti-immigrant political rhetoric. Recent work finds that some segments of Latinos (e.g., later generation, working class, and Protestant) supported Trump in the 2016 and 2020 elections (Corral and Leal, 2020; Fraga et al., 2025). Others find that more acculturated Latinos without discrimination experiences are more supportive of restrictive immigration policies (Pedraza, 2014), and Latinos who prioritize their American identity over their Latino identity or hold animus toward Latino immigrants are more supportive of anti-immigrant candidates and policies (Hickel Jr et al., 2020; Hickel Jr et al., 2024). Thus, whether native-born Latinos will punish a candidate who makes explicitly hostile remarks about immigrants as severely as foreign-born Latinos is not a foregone conclusion.

Our study also includes South Asian Canadians, the largest immigrant minority group in Canada, comprising about 6% of the population. This group makes up roughly 25% of the country’s visible minorities (non-white and non-Indigenous people). The category includes people with origins in India, Pakistan, Sri Lanka, and Bangladesh, with India accounting for the largest share. The South Asian population in Canada is highly diverse, with no single religion or language constituting a majority (Statistics Canada, 2022). Most South Asian Canadians are relatively recent arrivals, as large-scale non-European immigration did not begin until the 1970s (Triadafilopoulos, 2012). Today, about 70% of South Asian Canadians are immigrants, and nearly all are first- or second-generation (Statistics Canada, 2022).

South Asian Canadians are often viewed as politically successful and well integrated: they vote at higher rates than many other groups, express greater pride in the country (Soroka et al., 2008), and donate to political candidates at higher rates than Canadians of European origin (Besco and Tolley, 2022). Their success is also evident in elected office: after English and French, Punjabi is the third most common language spoken in the House of Commons (Rana, 2015), and in 2017, Jagmeet Singh—a Punjabi Sikh Canadian—became leader of the New Democratic Party, a well-established left-leaning party in Canada.

Compared to the United States, Canadian politics is generally less polarized along racial or immigration lines, though polarization has grown in recent years (Besco and Matthews, 2023). Politically, South Asians have historically leaned toward the Liberal Party—in recent elections more than 50% of South Asians voted Liberal, more than twice the share who supported the next place party (authors’ analysis of the Canadian Election Study).

When tensions arise in Canada, they are typically framed around immigration or refugee issues. When outgroups are negatively portrayed in media or political discourse, it is often Muslims—rather than South Asians more broadly—who bear the brunt of the backlash (Konitzer et al., 2019). Nonetheless, 72% of Canadians believe South Asian Canadians are often subject to racial discrimination (Neuman, 2025), and hate crimes targeting South Asians have increased in recent years (Liddar and Pallapothu, 2024).

Although Latinos in the U.S. and South Asians in Canada are both large immigrant-origin populations, there are important differences between them. South Asian Canadians make up a proportionately smaller share of the national population than Latinos in the U.S., and due to more recent migration histories, they are generally closer to the immigrant experience. Latino voter turnout surpassed 50% in the 2020 U.S. presidential election (NMAL 2025), but this is relatively low compared to South Asian Canadians, whose turnout in federal elections has been reported at nearly 90% (Statistics Canada 2022).

Moreover, while Latinos are frequently the explicit focus of anti-immigration rhetoric and policy in the U.S., political discourse in Canada rarely singles out South Asians. In the Canadian context, anti-immigrant sentiment more often targets other groups.^2^ Finally, immigration—and Latinos in particular—remain far more politically salient in the U.S. than either immigration or South Asians are in Canada.

These differences are methodologically useful: if we observe similar patterns of political response across distinct groups in contrasting national contexts, it strengthens the case for the generalizability of our study and its relevance beyond a single group or country.

## Data and Methods

To test our hypotheses we rely on two survey experiments: one conducted with a sample of 1,308 self-identified Latinos in the United States and the other with 815 South Asians in Canada. Both samples are broadly representative, though respondents tend to be somewhat older and more educated than the general population—a common feature of online samples that, as discussed below, appears to have little impact on the results. Respondents who completed the survey too quickly or provided nonsensical open-ended responses were replaced until target sample sizes were achieved.

Survey respondents were randomly assigned to one of three fictional campaign video conditions. To produce realistic campaign advertisements similar to those used by actual candidates, we purchased stock video, photos, and music, and hired a professional video editor along with voice actors for the narration. Each of the three one-minute campaign videos featured the same fictional white candidate “John Stevens,” and varied only in the central message: (1) the economy (control), (2) immigration, or (3) a panethnic appeal—targeting Latinos in the U.S. sample and South Asians in the Canadian sample.

After viewing their assigned video, respondents completed a series of post-treatment questions measuring emotional reactions and candidate evaluations. To preserve internal validity, the two treatment videos (immigrant and panethnic) explicitly referenced the target groups, while all other content was held constant. The immigrant version criticized immigration in general terms, without mentioning any specific racial or ethnic group. In contrast, the panethnic version targeted Latinos (in the U.S. study) or South Asians (in the Canadian study). The language across the two treatment conditions was nearly identical, with only the target group term (e.g., “immigrants” vs. “Latinos” or “South Asians”) and a few context-specific phrases modified to maintain coherence.^3^

Table 1 displays how the three different video ads (economic control, immigrant treatment, and panethnic treatment) are expected to target the different groups in the self-identifying U.S. Latino/Canadian South Asian samples. We also split the sample into respondents who were born abroad, and Latinos/South Asians who were born in the U.S./Canada, producing a total of six cells. Table 1 displays how these potential intersections impact the targeting within each treatment. Native-born Latinos/South Asians remain the neighboring group for the immigrant treatment, and the target group for the panethnic treatment, just like in Table 1. However, foreign-born Latinos/South Asians could be the target group for both the immigrant treatment *and* the panethnic treatment since they are factually immigrants (while at the same time identifying as Latinos/South Asians).

**Table 1.** Neighboring and Target Groups by Nativity Across Experimental Conditions.

	Economic Control	Immigrant Treatment	Panethnic Treatment
Native-born Latinos/South Asians	Not targeted	Neighboring group	Target group
Foreign-born Latinos/South Asians	Not targeted	Target group	Target group

Having two treatment conditions—immigrant and panethnic—allow us to causally compare responses between immigrant and native-born respondents. While we include statistical controls, unobserved differences between these groups may still exist. For example, if immigrants generally care more or less about politics, this could independently affect how they respond to political messages. If such baseline differences are driving the results, we would expect to observe divergent treatment effects between immigrants and non-immigrants even under the panethnic condition, where both groups are targeted. However, if both groups respond similarly to the panethnic treatment, this would suggest that any differential response to the immigrant treatment is attributable to the specific role of targeted versus neighboring group identity.

## Outcome Measures

We examine two primary sets of outcome measures: emotional responses to the ad and evaluations of the fictional candidate. Emotional responses are key mechanisms in political engagement, and eliciting specific emotions is often a central aim of campaign advertising (Weber 2013). In particular, negative ads that provoke fear or anger may disrupt a voter’s “standing choice,” prompting critical reflection and potentially shifting political attitudes (Brader 2005).

To measure emotional reactions, respondents were asked how the ad made them feel, using five specific emotions: three negative (sad, angry, and afraid) and two positive (enthusiastic and hopeful). While there is debate about the ability of survey respondents to accurately distinguish and report discrete emotional states (e.g., Larsen and Fredrickson, 1999; Wallbott and Scherer, 1989), we do not take a strong position on this issue. Our primary interest lies in capturing general positive versus negative emotional reactions. All emotional responses were measured using a 5-point Likert scale ranging from strong disagreement to strong agreement.

The second set of outcomes focused on candidate evaluations. Respondents rated the fictional candidate, John Stevens, on three character traits: honesty, work ethic, and whether he “cares about people like you,” and if they would vote for Stevens (yes/no).

In the analyses that follow, we regress these outcome variables on indicators for the immigrant and panethnic treatment conditions. To examine potential heterogeneity between native-born and immigrant respondents, we include controls for gender, education, income, age, language, country of origin, and political interest to adjust for demographic differences across subgroups. All outcome variables are rescaled to range from 0 to 1. Full model specifications and detailed results are available in Appendix B.

## Study 1: Latino Americans

We first present Average Treatment Effects (ATEs) of the emotional reaction outcomes among foreign-born respondents (always a direct target group) and native-born respondents (target group for the panethnic attack, neighboring group for the immigrant attack). All figures display ATEs with 95% confidence bands for native-born and foreign-born Latinos, with separate models for each dependent variable.

**Figure 1.**
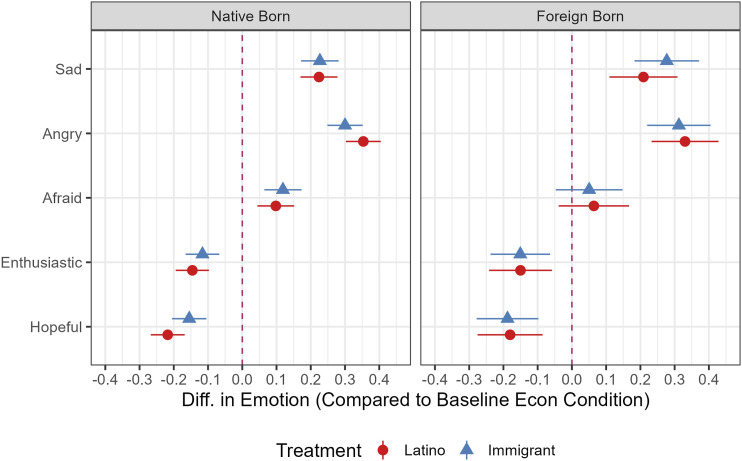
Emotion ATEs, Latino sample by nativity. *Note.* Average Treatment Effects with 95% CIs are derived from OLS regression results reported in Appendix Table 6 and 7. Models control for gender, education, income, age, country of origin, political interest, and English language. ATEs without control variables are substantively similar, as reported in the referenced Tables.

As Figure 1 shows, the treatment effects are in the expected direction, and statistically significant, confirming H1. Positive emotions (*enthusiasm* and *hope*) are lower in the treatment conditions, and negative emotions (*sadness, anger, fear*) are higher in the treatment than control conditions. The exception is *fear* for foreign-born respondents, which is in the expected direction but not statistically significant. The treatment effects are quite large.

The key comparison is between the panethnic and immigrant treatments for native-born respondents. For all the outcome measures related to emotions, native-born respondents react quite similarly, regardless of whether the politician is attacking Latinos in specific (a group they themselves belong to), or immigrants more broadly (a group they do not primarily belong to). This finding supports H3, the neighboring identities hypothesis.

**Figure 2.**
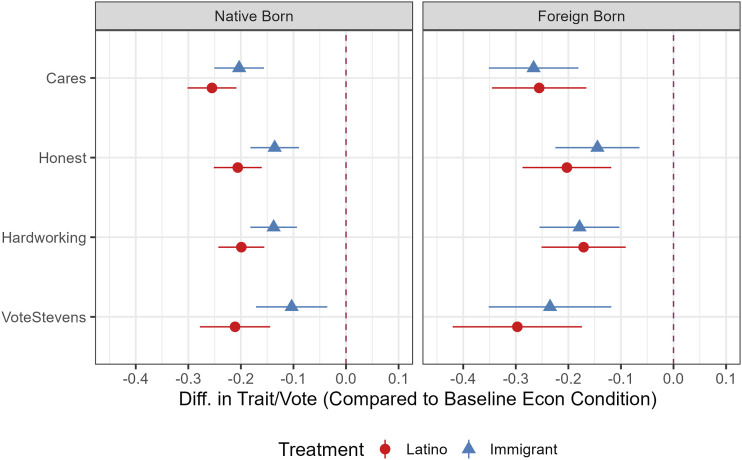
Candidate evaluation ATEs, Latino sample by nativity. *Note.* Average Treatment Effects with 95% CIs are derived from OLS regression results reported in Appendix Table 12 and 13. Models control for gender, education, income, age, country of origin, political interest, and English language. ATEs without control variables are substantively similar, as reported in the referenced Tables.

Next, we turn to candidate evaluations. The results in Figure 2 show uniformly negative results: when the candidate attacks Latinos in specific or immigrants more generally, he is evaluated much more negatively across all the measures (relative to the control condition). Again, the effects are quite large, all statistically significant, and in the expected direction.

There is some evidence that native-born Latino respondents distinguish between attacks on themselves as opposed to the neighboring (immigrant) group, with the panethnic treatment effects all somewhat larger than the immigrant treatment effects. Nonetheless, native-born respondents clearly react strongly to the anti-immigrant treatment. We interpret this finding as partial evidence of the neighboring identities hypothesis (H3), since non-members show large effects to attacks on neighboring groups, though not quite as large as those for attacks on themselves.

Finally, we examine a comparison between the two treatment conditions. The ATE here is the difference between the immigrant and panethnic treatments. This approach is useful for two reasons. First, it provides a simple measure of statistical significance between treatment effects. Second, despite including controls for various factors, it is possible that immigrant and non-immigrant respondents in the previous analysis were reacting differently to the economic control condition, and this is driving the results. Our expectation was small treatment effects for native-born respondents in the immigrant condition, but no differences for immigrants when comparing the panethnic treatment to the immigrant treatment.

**Figure 3.**
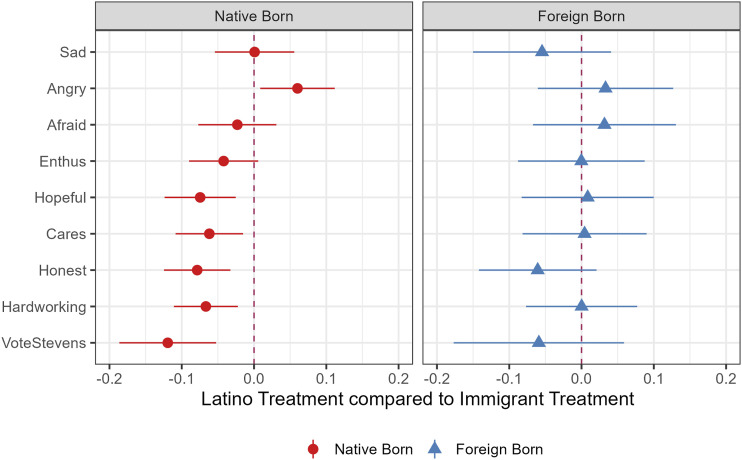
Difference between panethnic and immigrant treatments, Latino sample by nativity. *Note.* Difference between the two treatment conditions with 95% CIs. Negative values show the Latino treatment effect is larger than the immigrant treatment.

Figure 3 shows ATEs for the emotion and candidate evaluation outcome variables. For foreign-born (immigrant) respondents, there is no statistically significant difference between the panethnic and immigrant treatments on any measure. For native-born respondents, there is also no difference on any of the emotion variables except angry, which is in the opposite of the expected direction. However, for native-born respondents, all candidate evaluation treatments are negative and statistically significant, showing that the candidate was evaluated more negatively when attacks were directed specifically at their own group (Latinos) than when attacks reference immigrants in general. To be clear, this finding does not suggest that the message in the immigrant treatment is supported by native-born Latinos. Rather, it simply shows that the panethnic treatment may, in some cases, elicit somewhat stronger reactions than the immigrant treatment. Thus, although both membership and neighboring identities matter, the effects of membership may be somewhat stronger. Taking the evidence on emotions and candidate evaluations together, we interpret the Latino sample results to be partially supportive of the neighboring identity hypothesis (H3).

## Study 2: South Asian Canadians

Next we turn to Study 2, which replicates the experiment and analysis for South Asian Canadians. The procedures are essentially the same, with necessary country-specific adjustments to party and group names. As before, we first show the effects of the South Asian and immigrant treatments relative to the control (economy) treatment, and subsequently compare the two treatment conditions directly.

**Figure 4.**
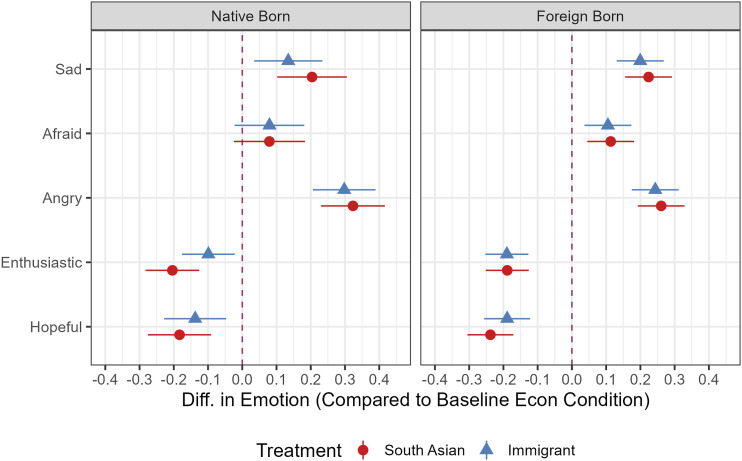
Emotion ATEs, South Asian sample by nativity. *Note.* Average Treatment Effects with 95% CIs are derived from OLS regression results reported in Appendix Table 9 and 10. Models control for gender, education, income, age, country of origin, political interest, and English language. ATEs without control variables are similar, see appendix.

Beginning with treatment effects compared to the economic control, the results are strikingly similar to those for Latinos in Study 1. As Figure 4 demonstrates, South Asians in either treatment arm reported lower positive emotions and higher negative emotions, compared to the control condition. The only exception is the outcome variable *afraid*, where the treatment effects for native-born respondents are not statistically significant, although they are in the expected direction. Crucially, the panethnic and immigrant treatment effects for native-born respondents have very similar ATEs across all measures, showing that target and neighboring group effects are quite similar to each other.

**Figure 5.**
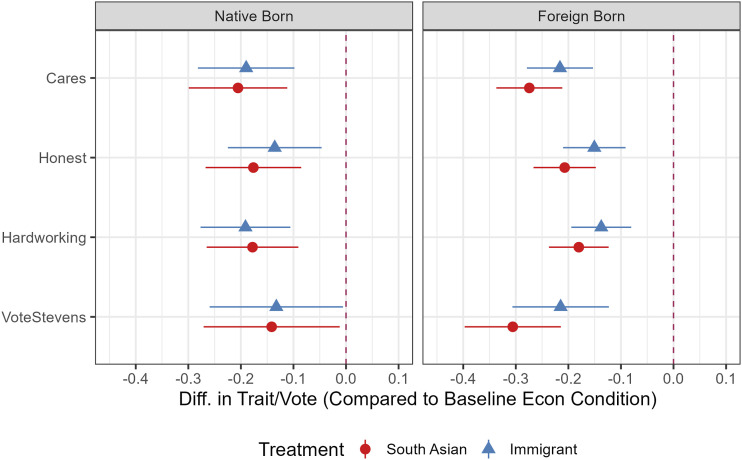
Candidate evaluation ATEs, South Asian sample by nativity. *Note.* Average Treatment Effects with 95% CIs are derived from OLS regression results reported in Table 15 and Table 16. Models control for gender, education, income, age, country of origin, political interest, and English language. ATEs without control variables are similar, see appendix.

Turning to candidate evaluations, Figure 5 presents average treatment effects (ATEs) across all measures: both treatments led native- and foreign-born respondents to evaluate the candidate more negatively relative to the economic control condition. The magnitude of these effects is substantial—averaging around 20 percentage points—especially among foreign-born respondents, though some variation exists across measures. Notably, native-born respondents exhibited nearly identical responses to the panethnic (target) and immigrant (neighboring) treatments, with overlapping confidence intervals indicating no meaningful difference between the two conditions.

**Figure 6.**
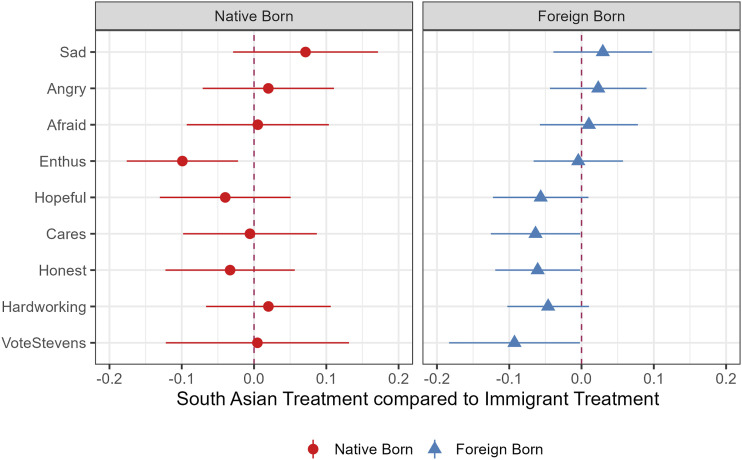
Difference between panethnic and immigrant treatments, South Asian sample by nativity. *Note.* Difference between the two treatment conditions with 95% CIs. Negative values show South Asian treatment effect is larger than the immigrant treatment.

Finally, we directly compare the panethnic treatment to the immigrant treatment. As shown in Figure 6, we find no significant differences among native-born South Asians, with the exception of the enthusiasm outcome. Among foreign-born South Asians—unlike foreign-born Latinos—the panethnic treatment produces somewhat stronger and statistically significant effects on 3 out of 9 outcome measures. However, this pattern does not offer clear support for or against the neighboring group hypothesis. Still, this result differs from the comparable Latino analysis in Figure 3, where the panethnic target effect is stronger than the immigrant neighboring effect. Taken together, these results of Study 2 provide more support for the neighboring identity hypothesis (H3) than those in Study 1.

In sum, we find clear and substantial treatment effects across both conditions, providing strong support for H1. Both U.S. Latinos and Canadian South Asians reported statistically and substantively significant emotional reactions and more negative evaluations of the candidate after viewing the attack ads, relative to those in the economic control group.

By contrast, the distinction between being in the targeted group versus the neighboring group yields relatively small and inconsistent differences. Contrary to H2, the panethnic treatment (a direct attack) does not consistently elicit stronger effects among native-born respondents compared to the immigrant treatment (an indirect attack). In instances where statistically significant differences do emerge, the gap in ATEs is modest—typically between 5 and 10 percentage points—and markedly smaller than the differences observed between either treatment and the control. Together, the overall pattern of results leads us to reject H2 in favor of H3.

## Additional Analysis and Robustness Checks

To assess the robustness of our results, we conducted additional analyses excluding respondents who failed the factual manipulation check, which asked participants to recall the topic of the video they viewed (see Appendix A.1). Approximately 10% of respondents in each sample did not pass this check. After removing these individuals, we re-estimated all models. The results remained unchanged: the average treatment effects (ATEs) were both statistically and substantively similar to the main analyses, as shown in Appendix Tables 17–24.

As noted earlier, we also tested treatment effects by nativity with and without control variables. These models are reported in the Appendix, beginning with Appendix Table 5, and show very similar results. Finally, we provide models with the full sample, as opposed to split by nativity, in Appendix Tables 6, 8, 11, and 14. All these models show that the panethnic and immigrant treatments produce statistically and substantively significant effects relative to the control condition.

## Discussion and Conclusion

When an identity group is attacked, its members often react strongly. But will those in a neighboring group respond just as forcefully? Our findings suggest they can: membership in a neighboring group can generate political responses that are comparable in strength to those of directly targeted group members.

Across two national contexts and two minority groups, we find that political attacks on immigrants provoke strong emotional reactions and negative candidate evaluations not only among immigrants themselves, but also among non-immigrant coethnics. Contrary to some expectations, non-immigrant respondents—who we conceptualized as a neighboring group—exhibited effects that were not only directionally similar but comparable in magnitude.

Because we used separate treatment conditions targeting immigrants and panethnic groups, these results are not confounded by differences in group composition or by disparities in partisanship or political attentiveness between immigrant and non-immigrant respondents. Even within each group, the distinction between being a member of the targeted group or the neighboring group produced little variation in treatment effects.

There is some indication that individuals can distinguish between being a target and being a neighboring group member. For instance, U.S.-born Latinos show somewhat smaller effects for attacks on immigrants than for attacks on Latinos. However, these differences are modest, appear only on select measures, and are overshadowed by the overall strength of the general treatment effects.

Traditional theories of social identity and policy threat suggest that members of directly targeted or threatened groups will respond most strongly. By contrast, theories of superordinate identity and proximal contact predict broader effects beyond the targeted group. What is particularly notable in our findings is that respondents are not members of the attacked group nevertheless exhibit reactions that are statistically and substantively similar to those of the targeted group. To our knowledge, prior research has not explored such effects among non-group members, nor do existing theories predict such equivalence in magnitude.

This research opens several pathways for future theoretical and empirical development. One possibility is that identification may be more ephemeral or metaphorical—yet no less meaningful—than traditionally conceptualized. Even individuals born in the United States or Canada may identify, in some sense, as immigrants, especially given the way they are often portrayed in public discourse and media narratives (Bedolla, 2005). Notably, researchers rarely measure the strength of group identity among non-members, yet multi-item identity scales reveal that non-immigrants often register non-zero levels of immigrant identity.^4^ Though difficult to disentangle this kind of identification from related constructs such as affect or perceived commonality given high correlations, measurement error, and ambiguous causal ordering, this area warrants further exploration, particularly in light of the continued use of explicit attacks on minority groups in contemporary politics.

A second avenue for future research concerns the causal mechanisms underlying neighboring group effects. For instance, responses may be shaped by social ties, perceived group commonality, or rational heuristics linking group threats to personal interests. The absence of a traditional identity mechanism does not imply a weaker response; instead, it may reflect the presence of alternative—but equally strong—motivating forces.

Of course, the strength of neighboring group effects is likely to vary depending on the groups and context involved. When social ties are weak or the perceived connection between groups is less apparent, responses among neighboring group members may be considerably smaller than those of directly targeted groups. What our findings demonstrate, however, is that such differentiation is not inevitable: in some cases, neighboring group identities can generate political reactions that are just as strong as those of targeted group members.

That we observe similar effects across distinct ethnic groups and national contexts speaks to the broader applicability of these findings. Recent anti-immigrant and anti-Latino rhetoric in the U.S. may have effectively “pre-treated” respondents, heightening sensitivity to political attacks. In contrast, Canada has experienced fewer instances of explicitly racist or anti-immigrant political discourse. While racism and discrimination persist, their political expression is markedly different. Moreover, the two groups in our study, Latinos in the U.S. and South Asians in Canada, differ in terms of immigration history, political incorporation, and broader societal positioning. Yet despite these differences, the results are remarkably consistent across both contexts. This consistency suggests that meaningful links exist between targeted and neighboring groups, and that strong political responses are not confined to narrowly defined group boundaries. The effects we document are not idiosyncratic to a particular ethnic group or national political system, but rather point to broader dynamics of group-based threat and solidarity.

Like all studies, ours is not without limitations. While we examine two distinct ethnic groups across two national contexts, our findings should not be presumed to generalize to all immigrant communities or minority groups. Additionally, the surveys relied on opt-in online samples conducted in English, which may underrepresent recent immigrants or individuals with lower levels of English proficiency and digital literacy. In the Canadian sample, we did not field a French-language version of the survey, resulting in very limited representation from Quebec, a province where, in any case, the South Asian population is relatively small (Statistics Canada, 2021). Moreover, our experimental treatments employed intentionally explicit political messages. It is possible that more subtle or ambiguous political attacks might produce weaker effects overall. That said, we have no specific reason to believe that the core findings would differ substantially across different target groups.

The implications for real-world politics are significant, particularly with respect to intergroup coalitions and cross-group solidarity. Attacks on minority groups remain distressingly common across many countries. Politicians may have incentives to target minorities—especially small, marginalized, or politically vulnerable groups—because such attacks can rally segments of the electorate while limiting the potential for effective backlash, or because some targets are more socially acceptable than others (Arora, 2025). In Europe, these attacks often focus on “migrants,” while in the United States, the rhetoric centers on “illegal immigrants.” In Canada, explicitly hostile rhetoric is less frequent but still surfaces in political discourse targeting Muslims (Salutin, 2024), temporary foreign workers (Tasker, 2024), and asylum seekers. Because targeted groups are likely to resist and mobilize, political actors may strategically aim their attacks at those perceived as least able to respond. However, our findings suggest this strategy is risky and may backfire. Even when the target is a numerically small or marginalized group, political attacks can activate broader neighboring group responses, leading to wider mobilization. This implies that native-born community members may oppose anti-immigrant politicians, other religious minorities may push back against anti-Muslim rhetoric, and immigrants more generally may object to attacks on specific migrant subgroups. Such dynamics have the potential to generate broad, multi-group coalitions in defense of shared principles or group-linked threats.

By highlighting the role of neighboring group dynamics, this research advances our understanding of how political attacks influence group behavior. Future work should investigate the conditions under which neighboring identities are activated and examine the implications of these dynamics for coalition-building, solidarity, and political participation. Our study also underscores the importance of exploring multiple identity in political behavior, particularly within an increasingly diverse and interconnected political landscape. If these dynamics are indeed generalizable, they carry far-reaching implications. They may help explain, for instance, how partisans in proportional representation systems react to criticisms of coalition partners, how citizens respond to attacks on neighboring countries, or how various social groups are embedded within overlapping and interconnected webs of identity, rather than occupying clearly bounded categories.

## Supplemental Material

Supplemental Material - Neighboring Groups and Political Attacks Supplemental Material for Neighboring Groups and Political Attacks by Randy Besco, Sergio Garcia-Rios, Julius Lagodny, Nazita, Lajevardi, Kassra Oskooii, and Erin Tolley in Political Research Quarterly.

## Notes

1. Here, we largely rely on the term “Latino,” but use the terms “Latino,” “Latinx,” and “Latine” interchangeably. These terms are fluid and reflect the evolving and inclusive ways in which individuals of Latin American heritage may identify. We acknowledge the importance of embracing these flexible terms as part of an ongoing conversation about identity and representation within the community.

2. For instance, debates over religious symbols in Quebec typically center on Muslims rather than Hindus, Sikhs, or specific ethnic groups. More recently, concerns over rising housing prices have been linked to international students—some of whom are South Asian—leading to incidents of antiIndian racism (Liddar and Pallapothu, 2024). However, mainstream political discourse continues to frame immigration issues broadly, without explicitly targeting South Asians (e.g., Tasker 2024).

3. Additional details about the samples and procedures, including question wording, respondent demographics, and treatments are in the appendix. Replication materials are available at https://doi.org/10.7910/DVN/UVJ6BB.

4. For example, see Appendix Figure A1, which shows that while immigrant identity is lower among native-born respondents than among immigrants, it is not zero. Because identity was measured post-treatment, we do not analyze treatment effects by immigrant identity in the main results.
