# Supplementary material for: Neighboring Groups and Political Attacks
Source: Polit Res Q. 2025 Aug 27;78(4):1474–90. doi: 10.1177/10659129251365875 (PMC12527296; doi:10.1177/10659129251365875)
Supplement: Supplemental Material - Neighboring Groups and Political Attacks [file sj-pdf-1-prq-10.1177_10659129251365875.pdf]

Appendix

Table of Contents

A Part I – Sample and Treatment Information **2**

A.1 Survey Sample Information 2

A.2 Ethics, IRB, and Debriefing 11

B Part II – Detailed Results for Main Models **13**

B.1 Emotion ATEs: Latinos 13

B.2 Emotion ATEs: Asians 15

B.3 Candidate Evaluation ATEs: Latinos 16

B.4 Candidate Evaluation ATEs: Asians 18

C Part III – Supplementary Models **19**

C.1 Emotion ATEs + Manipulation Check: Latinos 19

C.2 Emotion ATEs + Manipulation Check: Asians 20

C.3 Candidate Evaluation ATEs + Manipulation Check: Latinos 21

C.4 Candidate Evaluation ATEs + Manipulation Check: Asians 22

C.5 Immigrant Identity 23

A Part I – Sample and Treatment Information

A.1 Survey Sample Information

The South Asian survey was fielded in Canada in June 2019, with the sample provided by Qualtrics. The Latino sample was provided by Dynata (formerly SSI) and fielded in July 2019.

In both surveys only self-identifying Latino and South Asian respondents were permitted to take the study. We also imposed demographic quotas: both surveys needed to contain at least 30% native-born respondents and a balanced gender quota. No respondents said they were under 18, and we also checked IP addresses for location (country).

Respondents who provided nonsensical responses to open-ended questions or took less than 1/2 the median time were dropped and replaced by the survey company. In addition, we included a factual manipulation check and, while we did not drop respondents based on this criterion, 90 percent of respondents in the panel passed. The question for the manipulation check was; 'A few minutes ago you were shown a video about a candidate. Do you remember what the video was about?' The answer options were 'Immigrants are a problem' (1), 'Need to improve the economy' (2), 'Climate change is a serious threat' (3), 'Latinos/South Asians are a problem' (4), or 'I didn't see a video' (5), with correct response depending on the condition.

Table 1: American Latino Population and Sample Demographic Characteristics

	**Population**	**Sample**
Income (Median)	$55,321	$40,000-59,999
Bachelor's Degree or higher	21%	38%
Age (Median)	30	48
Born in the US	65%	74%
Gender(Women)	48%	53%
Mexican	61%	47%
Puerto Rican	9.6%	21%
Cuban	3.9%	10%
Dominican	3.9%	3%

Note: These figures come from the US Census, Pew Research Center, and the US Department of Health and Human Services, Office of Minority Health.

Table 2: Canadian South-Asian Population and Sample Demographic Characteristics

	Population	Sample
Income (Median) (CAD)	$81,453	$60,000 - $79,999
Bachelor's Degree or higher	38.3%	71.0%
Age (Median)	33	32
Born in Canada	30.9%	30.0%
Gender (Women)	49.2%	55.0%
Indian	80.6%	65.3%
Pakistani	11.0%	8.2%
Bangladeshi	2.3%	4.5%
Sri Lankan	7.8%	12.6%

Note: Percentages may not add to 100 since only selected groups are included. These figures come from Statistics Canada tables 98-400-X2016192, 98-404-X2016001, 98-400-X2016192, 98-400-X2016192, 98-400-X2016187, and from the Statistics Canada report "Housing experiences in Canada: South Asian people in 2018"

Table 3: Latino Sample Balance Table

Variable	Control	Ethnicity	Immigrant
Age	41.80	42.64	43.27
Income	3.11	3.08	3.19
Education	5.65	5.64	5.62
Female	0.54	0.47	0.53
Native Born	0.76	0.77	0.72
N	441.00	430.00	437.00

Table 4: South Asian Balance Table

Variable	Control	Ethnicity	Immigrant
Age	38.17	38.08	38.62
Income	3.54	3.53	3.54
Education	4.50	4.37	4.47
Female	0.55	0.53	0.49
Native Born	0.30	0.29	0.31
N	264.00	273.00	278.00

Video Scripts and Links

The campaign script drew heavily on language from actual political advertisements, speeches, and media coverage. This approach served two purposes. First, incorporating familiar phrases and tropes enhanced the realism of the experiment and increased its external validity, as respondents were exposed to rhetoric they might plausibly encounter in real-world politics. Second, for ethical reasons, we sought to avoid introducing new or fabricated offensive content into participants' information environments. To further mitigate potential harm, we refrained from referencing real politicians, electoral districts, public offices, or political parties. At the conclusion of the survey, all respondents received a debriefing with accurate information about the issues referenced in the video conditions.[6]

The campaign treatments referred to immigrants or Latinos/South Asians who "keep coming, thousands every year," and accused too many members of the referenced group of being on "welfare." The ads noted how "crime is rising."[7] These phrases and terms are similar to those used by various candidates, including Donald Trump and Ed Gillespie, a 2017 candidate for Governor of Virginia, whose anti-Latino ads received widespread media coverage. The video about the economy used standard political tropes about job creation and improving the economy, and the candidate emphasized the need to "create new jobs" to "kick-start the economy." The video also included intentionally negative content (e.g., implying the economy is bad, ominous background music) to match the negativity of the treatment videos and avoid effects based on the tone of the videos rather than their specific content. Finally, we included this control condition instead of an empty control condition/no video because watching a campaign ad of any kind might stimulate engagement. In addition, asking questions about a candidate without providing any information at all would likely have lacked face validity.[8]

Scripts for treatment videos are shown below. The videos are only in English, which has some limitations but ultimately chose to do so given respondents would likely not believe an anti-Latino ad aired in Spanish. The notes indicate the general sources and context of the phrases used. The script was written after a review of real speeches, advertisements, and media coverage of related issues, drawing on commonly used phrases and key terms. Because we plan to run the experiment in other countries, the treatments intentionally include language that would be recognizable in multiple contexts. We also produced two orthogonal party conditions (Republican/No Party), but since this is not of theoretical interest here we collapse

6 The research design received institutional review board approval at the University of Toronto. See debriefing statement in Appendix A.2.

7 The the full text, links to videos, and description of the sourcing of phrases is available in Appendix A.1.

8 A pilot study did include a pure control, but we did not adopt this approach for the main survey because a video of any kind increased political engagement (which we examine in a separate paper). We also produced orthogonal party conditions (Republican/Conservative or no party mention), but did not find statistically significant treatment effects by party. Since this is not of theoretical interest here, we collapse the cells to maximize power. We also tested the treatments with a left-wing party condition (Democrat/Liberal) in a pilot. However, questions were raised about the face validity of a left-wing anti-immigrant candidate, so we did not employ this approach in the study.

the cells. We also included a Democratic treatment in a pilot study, but did not proceed with it in the current study, since several respondents suggested an explicitly racist or anti-immigrant Democrat lacked face validity.

The videos can be found here:

- https://www.dropbox.com/sh/oh6lc26qz96wipa/AAA6tA8dpY9hHTEuU9UN-zCQa?dl=0, Password: USVideos

Immigrant Treatment Script

Woman: Sometimes I look around, and I don't recognize my own community[9]. Immigrants, they don't speak English[10], they don't understand how we do things here.

Man Narrator: Immigrants are flooding in. Thousands ever year. They don't have any documents, but just say "asylum"[11], and they are let in.

Man Narrator: Now costs are skyrocketing. Too many immigrants are on welfare[12]. They're collecting free benefits that should be going to hardworking, taxpaying, Americans.

Man Narrator: And crime is rising. Break-ins. Knife attacks[13]. Immigrant gangs[14].

Woman: "I don't want to be a foreigner in my own country. We need to do something now.

Man Narrator: John Stevens will protect our communities, and get immigration under control.

John Stevens: Its time to secure our borders[15]. If these people don't accept our values[16], we should send them back[17]. We need to focus on helping ordinary, hardworking Americans. This is our country, and we're taking it back[18].

Man Narrator: Vote for Republican/no party John Stevens. The Candidate for Real Americans.

[9] Used in Brexit UK media coverage
[10] Stereotype employed in multiple countries
[11] Terms in used in UK and Germany, increasingly in US.
[12] Common US phrase, also used in speech by Germany AfD politician.
[13] Issue in UK and Germany
[14] Referenced in US campaign ads (MS13 etc.)
[15] Used by both US and Canadian politicians
[16] Used by candidate for Canadian Conservative party, also common in Germany
[17] Used in Trump ads
[18] Used in Trump ads

Latino Treatment Script

Woman: Sometimes I look around, and I don't recognize my own community. Hispanics, they don't speak English, they don't understand how we do things here.

Man Narrator: Now costs are skyrocketing. Too many Hispanics are on welfare. They're collecting free benefits that should be going to hardworking, taxpaying, Americans.

Man Narrator: And crime is rising. Break-ins. Knife attacks. Latino/South Asians gangs.

Woman: This has to stop. We need to do something now.

Man Narrator: John Stevens will protect our communities.

John Stevens: We need to focus on helping ordinary, hardworking Americans. People who share our values. This is our country, and we're taking it back.

Man Narrator: Vote for Republican/no party John Stevens. The Candidate for Real Americans.

Economy (Control) Script

Woman: Things have been so bad these last few years, and prices keep going up. I want to give my kids a good life, but it's hard to make ends meet. It's been really tough on us.

Man Narrator: We need to protect our economy. Even if you can have a job, you never know if it's going to last. It's harder to make ends meet. Debts keep piling up. Taxes are higher. Young people can't find jobs, and when they do, those jobs pays less.

Woman: This has to stop. We need to do something now.

Man Narrator: John Stevens will protect our economy and bring in new jobs.

John Stevens: We need to focus on helping everyday Americans. People who work hard deserve good paying jobs. It's time to kick-start the economy and get Americans back to work.

Man Narrator: Vote for Republican/no party John Stevens. The Candidate for Jobs

Question Wording

What is your gender?

- Man
- Woman
- Other

What racial or ethnic group best describes you? *Note: used as filter question, only respondents who selected Hispanic or Latino continued. American survey only*

- White
- Hispanic or Latino
- Black or African-American
- Asian or Asian-American
- Native American
- Middle Eastern
- Other [open text]

Hispanics and Latinos have their roots in many different place in Latin America. Thinking about your family origins, are you (select all that apply): *Note: American survey only*

- Mexican
- Colombian
- Guatemalan
- Dominican
- Salvadoran
- Cuban
- Puerto Rican
- Other [open text]

Statistics Canada uses the following racial or ethnic categories. Which one best describes you? *Note: used as filter question, only respondents who selected South Asian continued. Canadian survey only*

- South Asian
- Chinese
- Black
- Filipino
- Latin American

- Arab
- Southeast Asian
- West Asian
- Korean
- Japanese
- Indigenous
- White
- Other

What year were you born?
What is the highest level of education you have completed? *Note: American survey only*

- Did not graduate from high school
- High school graduate
- Some college, but no degree
- 2-year college degree
- 4-year college degree
- Postgraduate degree (MA, MBA, MD, JD, PhD, etc.)

What is the highest level of education you have completed? *Note: Canadian survey only*

- Did not graduate from high school
- High school graduate
- Some college, but no degree
- College degree
- University undergraduate degree
- Postgraduate degree (MA, MBA, MD, JD, PhD, etc.)

Thinking back over the last year, what was your household's total income before taxes? This question is completely confidential and just used to help classify the responses, but it is very important to the research.

- Less than $20,000
- $20,000 - $39,999
- $40,000 - $59,999

- $60,000 -$79,999
- $80,000 - $99,999
- $100,000 - $149,999
- $150,000 - $199,999
- $200,000 or more
- Don't know/Won't say

Which language are you most comfortable with? *Note: American survey only*

- English
- Spanish
- Other (textbox)

What languages do you speak? (check all that apply) *Note: Canadian survey only*

- English
- French
- Hindi
- Gujarati
- Punjabi
- Urdu
- Tamil
- Other (textbox)

A few minutes ago you were shown a video about a candidate. Do you remember what the video was about? *Note: American survey only*

- Immigrants are a problem
- Need to improve the economy
- Climate change is a serious threat
- Latinos are a problem
- I didn't see a video

Note: Coded 0-1, correct or incorrect response

A few minutes ago you were shown a video about a candidate. Do you remember what the video was about? *Note: Canadian survey only*

- Immigrants are a problem

- Need to improve the economy
- Climate change is a serious threat
- South Asians are a problem
- I didn't see a video

Note: Coded 0-1, correct or incorrect response
Did it make you feel: Sad?

- Scale from 5 to 1, ends labeled Very Sad and Not Sad at all

Did it make you feel: Angry?

- Scale from 5 to 1, ends labeled Very Angry and Not Angry at all

Did it make you feel: Enthusiastic?

- Scale from 5 to 1, ends labeled Very Enthusiastic and Not Enthusiastic at all

Did it make you feel: Afraid?

- Scale from 5 to 1, ends labeled Very Afraid and Not Afraid at all

Did it make you feel: Hopeful?

- Scale from 5 to 1, ends labeled Very Hopeful and Not Hopeful at all

Would you vote for candidate John Stevens?

- Yes
- No

Do you think that candidate John Stevens is honest?

- Scale from 0 to 10

Do you think that candidate John Stevens is hardworking?

- Scale from 0 to 10

Do you think that candidate John Stevens cares about people like you?

- Scale from 0 to 10

A.2 Ethics, IRB, and Debriefing

Ethics and Ethical Approval

This study received IRB approval from the University of Toronto's human participants ethnics protocol panel in April 2019.

The content of the video treatments is sensitive, offensive, and discriminatory. As such, the question of ethics is very important to detail and address. One of the overarching purposes of this study is to assess how minorities respond to an increasingly explicitly racist political environment. Politicians across western democracies are increasingly making derisive comments and appeals about immigrant and racial minorities, and these appeals are not heard in a vacuum. Rather, the targets of these appeals are also being exposed to such rhetoric, and it begs the question how political discrimination of this nature has affected these targeted populations.

As such, in developing the treatments, we took great care in ensuring a) that we did not introduce new harmful material or remarks into the information environment, and b) that we fully debriefed our respondents.

With respect to the first concern, the content of our treatments mirror real remarks made by politicians, including Donald Trump and Ed Gillespie, a 2017 candidate for Governor of Virginia, whose anti-Latino ads received widespread media coverage. The video about the economy used standard political tropes about job creation and improving the economy, and the candidate emphasized the need to "create new jobs" to "kick-start the economy." The video also included intentionally negative content (e.g., implying the economy is bad, ominous background music) to match the negativity of the treatment videos and avoid effects based on the tone of the videos rather than their specific content.

Per the second concern, we fully debriefed respondents upon the conclusion of the survey. The exact text we presented respondents with can be found below:

Text for Debriefing

> "Thank you very much for taking the survey! A fictional advertisement by a fictional politician was used as part of the survey: John Stevens is not running for office, and the ad is not real. However, words and phrases in the ad were taken from real speeches, political advertisements, and media coverage – but not necessarily in your country or by a particular candidate or party. Importantly, you should know that, in general, crime rates have been declining for many years, and immigrants actually commit less crimes than other people. The reason we used the ad is because it is important to understand how people in different countries react to these kinds of statements. Using a fictional ad helps separate feelings toward specific politicians from the things they say. We also didn't want to use the name of a real person, in case it creates confusion about what they actually said. This research is about many different kinds of political attitudes, but primarily about how anti-immigrant statements by politicians affect participation in politics. For example, we want to find out if it makes people get motivated and involved in politics, or if they makes them discouraged and uninterested in politics. We would ask you to maintain confidentiality about the survey, since any knowledge of the purpose by other respondents could bias the data and cause problems for the research. That might mean less surveys are done in the future. If you are interested in seeing the results after they have been analyzed, the researchers plan to use this data to publish academic research, and present at conferences. Among other places, when available this research will be posted at (X). If you have ethical concerns about the research (such as the way you have been treated or your rights as a participant), you may contact the Office of Research Ethics at X, or the researcher Professor X. If you have questions about the survey or the research, you may contact Professor X. If you are interested in these areas of research, you may wish to read the following articles: https://www.pewhispanic.org/2018/10/25/latinos-and-discrimination, https://docs.wixstatic.com/ugd/f1a7db_65c8b9304610496180dccc5809937776.pdf."

As can be seen, the text informs respondents that the video they just watched was not real and that John Stevens is not running for office. It corrects misinformation in the ad about increasing crime rates, and then proceeds to explain why we are conducting this study. Specifically, it explains that we are interested in understanding how the public respondents to such denigrating statements made by politicians. It also provides the contact information for the lead researcher and the ethics office at the lead researcher's institution where IRB was procured.

B Part II – Detailed Results for Main Models

B.1 Emotion ATEs: Latinos

Table 5: Emotions ATEs (Full Latino Sample)

	**Sad** Model 1	**Angry** Model 2	**Afraid** Model 3	**Enthusiastic** Model 4	**Hopeful** Model 5
Latino Treatment	0.221^{***}	0.349^{***}	0.090^{***}	-0.147^{***}	-0.209^{***}
	(0.024)	(0.023)	(0.024)	(0.022)	(0.022)
Immigrant Treatment	0.240^{***}	0.301^{***}	0.101^{***}	-0.125^{***}	-0.163^{***}
	(0.024)	(0.023)	(0.024)	(0.022)	(0.022)
Constant	0.423^{***}	0.361^{***}	0.312^{***}	0.373^{***}	0.448^{***}
	(0.017)	(0.016)	(0.017)	(0.015)	(0.016)
N	1308	1308	1308	1308	1308
R-squared	0.086	0.172	0.016	0.039	0.069
Adj. R-squared	0.085	0.170	0.014	0.038	0.068

$^{***}p < .01$; $^{**}p < .05$; $^{*}p < .1$

Table 6: Emotion ATEs (Native Born Latinos)

	**Sad**	**Sad**	**Angry**	**Angry**	**Afraid**	**Afraid**	**Enthus**	**Enthus**	**Hopeful**	**Hopeful**
	**Model 1**	**Model 2**	**Model 3**	**Model 4**	**Model 5**	**Model 6**	**Model 7**	**Model 8**	**Model 9**	**Model 10**
Latino Treat.	0.224***	0.227***	0.354***	0.360***	0.098***	0.098***	−0.146***	−0.152***	−0.218***	−0.224***
	(0.028)	(0.027)	(0.026)	(0.026)	(0.027)	(0.027)	(0.025)	(0.024)	(0.025)	(0.025)
Immigrant Treat.	0.227***	0.226***	0.300***	0.300***	0.119***	0.121***	−0.116***	−0.110***	−0.155***	−0.149***
	(0.028)	(0.028)	(0.026)	(0.026)	(0.028)	(0.027)	(0.025)	(0.024)	(0.026)	(0.025)
Female		0.049**		0.079***		0.038*		−0.062***		−0.044**
		(0.023)		(0.022)		(0.023)		(0.020)		(0.021)
Education		0.005		0.009		0.005		−0.015**		−0.008
		(0.008)		(0.007)		(0.008)		(0.007)		(0.007)
Income		−0.018*		−0.001		−0.007		−0.014		−0.014
		(0.010)		(0.009)		(0.010)		(0.009)		(0.009)
Age		0.001		0.001		−0.002**		−0.003***		−0.004***
		(0.001)		(0.001)		(0.001)		(0.001)		(0.001)
Mexican		−0.011		0.002		−0.054**		−0.027		−0.033
		(0.023)		(0.022)		(0.023)		(0.020)		(0.021)
English L.		−0.045		−0.039		−0.087***		−0.110***		−0.100***
		(0.033)		(0.031)		(0.032)		(0.029)		(0.030)
Pol. Interest		0.009**		0.008*		0.017***		0.018***		0.013***
		(0.004)		(0.004)		(0.004)		(0.004)		(0.004)
Constant	0.427***	0.362***	0.376***	0.231***	0.303***	0.343***	0.370***	0.662***	0.441***	0.725***
	(0.019)	(0.062)	(0.018)	(0.058)	(0.019)	(0.061)	(0.017)	(0.054)	(0.018)	(0.056)
N	984	984	984	984	984	984	984	984	984	984
R-squared	0.083	0.099	0.180	0.200	0.021	0.056	0.038	0.112	0.075	0.131
Adj. R-squared	0.081	0.091	0.179	0.193	0.019	0.047	0.036	0.104	0.073	0.123

***p < .01; **p < .05; *p < .1

Table 7: Emotion ATEs (Foreign Born Latinos)

	**Sad**	**Sad**	**Angry**	**Angry**	**Afraid**	**Afraid**	**Enthus**	**Enthus**	**Hopeful**	**Hopeful**
	**Model 1**	**Model 2**	**Model 3**	**Model 4**	**Model 5**	**Model 6**	**Model 7**	**Model 8**	**Model 9**	**Model 10**
Latino Treat.	0.209***	0.217***	0.330***	0.338***	0.064	0.074	−0.150***	−0.161***	−0.180***	−0.184***
	(0.051)	(0.050)	(0.050)	(0.049)	(0.052)	(0.052)	(0.047)	(0.046)	(0.048)	(0.048)
Immigrant Treat.	0.277***	0.271***	0.313***	0.305***	0.050	0.042	−0.151***	−0.161***	−0.188***	−0.193***
	(0.048)	(0.047)	(0.047)	(0.046)	(0.050)	(0.049)	(0.044)	(0.043)	(0.046)	(0.045)
Female		0.072*		0.021		0.039		−0.039		0.036
		(0.041)		(0.040)		(0.042)		(0.038)		(0.039)
Education		−0.016		−0.008		0.002		−0.003		−0.017
		(0.012)		(0.012)		(0.013)		(0.011)		(0.012)
Income		0.012		0.016		0.001		−0.008		−0.002
		(0.016)		(0.016)		(0.017)		(0.015)		(0.015)
Age		0.0001		−0.0004		−0.002		−0.001		−0.002
		(0.001)		(0.001)		(0.001)		(0.001)		(0.001)
Mexican		0.118**		0.156***		0.060		−0.084*		−0.104**
		(0.049)		(0.048)		(0.051)		(0.045)		(0.047)
English L.		−0.045		0.013		−0.064		−0.023		−0.021
		(0.040)		(0.039)		(0.041)		(0.037)		(0.038)
Pol. Interest		0.015**		0.011*		0.022***		0.026***		0.021***
		(0.007)		(0.007)		(0.007)		(0.006)		(0.007)
Constant	0.413***	0.319***	0.313***	0.197*	0.339***	0.261**	0.382***	0.367***	0.469***	0.539***
	(0.035)	(0.109)	(0.035)	(0.107)	(0.036)	(0.113)	(0.032)	(0.100)	(0.034)	(0.104)
N	324	324	324	324	324	324	324	324	324	324
R-squared	0.099	0.147	0.154	0.200	0.005	0.054	0.043	0.102	0.060	0.107
Adj. R-squared	0.093	0.122	0.149	0.177	−0.001	0.027	0.037	0.076	0.054	0.081

***p < .01; **p < .05; *p < .1

B.2 Emotion ATEs: Asians

Table 8: Emotions ATEs (Full Asian Sample)

	Sad<br>Model 1	Angry<br>Model 2	Afraid<br>Model 3	Enthusiastic<br>Model 4	Hopeful<br>Model 5
South Asian Treatment	0.218***	0.279***	0.103***	−0.192***	−0.221***
	(0.029)	(0.028)	(0.029)	(0.026)	(0.028)
Immigrant Treatment	0.179***	0.261***	0.098***	−0.163***	−0.175***
	(0.029)	(0.028)	(0.029)	(0.026)	(0.028)
Constant	0.443***	0.349***	0.377***	0.421***	0.485***
	(0.021)	(0.020)	(0.021)	(0.018)	(0.020)
N	815	815	815	815	815
R-squared	0.074	0.129	0.019	0.074	0.079
Adj. R-squared	0.072	0.127	0.017	0.071	0.077

$^{***}p < .01$; $^{**}p < .05$; $^{*}p < .1$

Table 9: Emotion ATEs (Native Born Asians)

	Sad<br>Model 1	Sad<br>Model 2	Angry<br>Model 3	Angry<br>Model 4	Afraid<br>Model 5	Afraid<br>Model 6	Enthus<br>Model 7	Enthus<br>Model 8	Hopeful<br>Model 9	Hopeful<br>Model 10
South Asian Treat.	0.204***	0.207***	0.323***	0.330***	0.079	0.088*	−0.204***	−0.204***	−0.183***	−0.181***
	(0.052)	(0.052)	(0.047)	(0.047)	(0.053)	(0.051)	(0.040)	(0.040)	(0.047)	(0.047)
Immigrant Treat.	0.134***	0.135***	0.298***	0.310***	0.079	0.083	−0.099**	−0.105***	−0.138***	−0.141***
	(0.051)	(0.051)	(0.046)	(0.047)	(0.052)	(0.050)	(0.039)	(0.039)	(0.046)	(0.046)
Female		0.060		0.095**		0.150***		−0.030		−0.049
		(0.043)		(0.039)		(0.042)		(0.033)		(0.039)
Education		0.018		0.023		0.052***		−0.011		−0.002
		(0.017)		(0.016)		(0.017)		(0.013)		(0.016)
Income		0.016		0.019		−0.003		−0.005		0.010
		(0.019)		(0.017)		(0.019)		(0.015)		(0.017)
Age		0.001		−0.003		−0.001		−0.0004		−0.001
		(0.002)		(0.002)		(0.002)		(0.002)		(0.002)
Indian		0.019		0.003		0.005		0.019		0.039
		(0.047)		(0.042)		(0.046)		(0.036)		(0.042)
English L.		−0.062		−0.048		−0.172**		−0.085		−0.104
		(0.071)		(0.065)		(0.070)		(0.055)		(0.064)
Pol. Interest		−0.003		−0.006		0.003		0.012*		0.010
		(0.008)		(0.008)		(0.008)		(0.006)		(0.008)
Constant	0.446***	0.300**	0.386***	0.340***	0.418***	0.284**	0.326***	0.410***	0.402***	0.441***
	(0.037)	(0.127)	(0.034)	(0.115)	(0.037)	(0.124)	(0.028)	(0.097)	(0.033)	(0.114)
N	246	246	246	246	246	246	246	246	246	246
R-squared	0.062	0.084	0.194	0.230	0.012	0.110	0.097	0.130	0.064	0.099
Adj. R-squared	0.055	0.050	0.187	0.200	0.004	0.076	0.090	0.097	0.056	0.064

$^{***}p < .01$; $^{**}p < .05$; $^{*}p < .1$

Table 10: Emotion ATEs (Foreign Born Asians)

	Sad<br>Model 1	Sad<br>Model 2	Angry<br>Model 3	Angry<br>Model 4	Afraid<br>Model 5	Afraid<br>Model 6	Enthus<br>Model 7	Enthus<br>Model 8	Hopeful<br>Model 9	Hopeful<br>Model 10
South Asian Treat.	0.224***	0.229***	0.261***	0.267***	0.113***	0.120***	−0.189***	−0.185***	−0.238***	−0.238***
	(0.035)	(0.035)	(0.035)	(0.034)	(0.035)	(0.035)	(0.032)	(0.032)	(0.034)	(0.034)
Immigrant Treat.	0.199***	0.200***	0.243***	0.244***	0.105***	0.110***	−0.190***	−0.181***	−0.189***	−0.181***
	(0.035)	(0.035)	(0.035)	(0.034)	(0.035)	(0.035)	(0.032)	(0.032)	(0.034)	(0.034)
Female		0.025		0.047		0.053*		0.002		−0.007
		(0.029)		(0.029)		(0.029)		(0.027)		(0.028)
Education		0.012		−0.001		0.029**		0.019*		0.003
		(0.011)		(0.011)		(0.011)		(0.010)		(0.011)
Income		0.006		0.063***		0.001		−0.027**		−0.038***
		(0.014)		(0.013)		(0.013)		(0.012)		(0.013)
Age		0.001		−0.0001		−0.001		−0.001		0.0004
		(0.001)		(0.001)		(0.001)		(0.001)		(0.001)
Indian		−0.001		−0.021		−0.015		0.023		0.068**
		(0.031)		(0.030)		(0.030)		(0.028)		(0.030)
English L.		0.015		0.025		−0.004		−0.090***		−0.090***
		(0.031)		(0.030)		(0.031)		(0.028)		(0.030)
Pol. Interest		0.007		0.009*		0.005		0.010*		0.003
		(0.006)		(0.006)		(0.006)		(0.005)		(0.006)
Constant	0.442***	0.253***	0.334***	0.028	0.359***	0.215**	0.462***	0.482***	0.520***	0.625***
	(0.025)	(0.084)	(0.025)	(0.082)	(0.025)	(0.084)	(0.023)	(0.076)	(0.024)	(0.082)
N	569	569	569	569	569	569	569	569	569	569
R-squared	0.080	0.090	0.110	0.156	0.023	0.043	0.076	0.111	0.087	0.120
Adj. R-squared	0.077	0.075	0.107	0.142	0.019	0.027	0.073	0.097	0.084	0.106

$^{***}p < .01$; $^{**}p < .05$; $^{*}p < .1$

B.3 Candidate Evaluation ATEs: Latinos

Table 11: Candidate Evaluation ATEs (Full Latino Sample)

	**Cares**<br>**Model 1**	**Honest**<br>**Model 2**	**Hardworking**<br>**Model 3**	**Vote Stevens**<br>**Model 4**
Latino Treatment	−0.256***	−0.206***	−0.193***	−0.231***
	(0.021)	(0.020)	(0.020)	(0.030)
Immigrant Treatment	−0.218***	−0.136***	−0.146***	−0.136***
	(0.021)	(0.020)	(0.020)	(0.030)
Constant	0.523***	0.539***	0.572***	0.406***
	(0.015)	(0.014)	(0.014)	(0.021)
N	1308	1308	1308	1308
R-squared	0.116	0.074	0.075	0.044
Adj. R-squared	0.115	0.073	0.074	0.043

***p < .01; **p < .05; *p < .1

Table 12: Candidate Evaluation ATEs (Native Born Latinos)

	**Cares**<br>**Model 1**	**Cares**<br>**Model 2**	**Honest**<br>**Model 3**	**Honest**<br>**Model 4**	**Hardworking**<br>**Model 5**	**Hardworking**<br>**Model 6**	**Vote**<br>**Model 7**	**Vote**<br>**Model 8**
Latino Treat.	−0.255***	−0.263***	−0.206***	−0.211***	−0.199***	−0.204***	−0.211***	−0.221***
	(0.024)	(0.023)	(0.023)	(0.023)	(0.022)	(0.022)	(0.034)	(0.033)
Immigrant Treat.	−0.203***	−0.201***	−0.136***	−0.133***	−0.138***	−0.138***	−0.103***	−0.101***
	(0.024)	(0.024)	(0.024)	(0.023)	(0.023)	(0.022)	(0.035)	(0.034)
Female		−0.083***		−0.038*		−0.041**		−0.080***
		(0.020)		(0.019)		(0.019)		(0.028)
Education		−0.009		−0.005		−0.006		−0.026***
		(0.007)		(0.007)		(0.006)		(0.010)
Income		−0.009		−0.004		−0.005		0.010
		(0.008)		(0.008)		(0.008)		(0.012)
Age		−0.002***		−0.002***		−0.0004		−0.004***
		(0.001)		(0.001)		(0.001)		(0.001)
Mexican		−0.032		−0.045**		−0.046**		−0.078***
		(0.020)		(0.019)		(0.018)		(0.028)
English L.		−0.072***		−0.046*		−0.026		−0.069*
		(0.028)		(0.028)		(0.027)		(0.040)
Pol. Interest		0.009**		0.012***		0.012***		0.014***
		(0.004)		(0.004)		(0.003)		(0.005)
Constant	0.507***	0.729***	0.525***	0.669***	0.556***	0.609***	0.383***	0.715***
	(0.017)	(0.053)	(0.016)	(0.052)	(0.016)	(0.050)	(0.024)	(0.076)
N	984	984	984	984	984	984	984	984
R-squared	0.116	0.155	0.077	0.109	0.079	0.103	0.038	0.082
Adj. R-squared	0.114	0.147	0.075	0.101	0.077	0.095	0.036	0.074

***p < .01; **p < .05; *p < .1

Table 13: Candidate Evaluation ATEs (Foreign Born Latinos)

	**Cares**	**Cares**	**Honest**	**Honest**	**Hardworking**	**Hardworking**	**Vote**	**Vote**
	**Model 1**	**Model 2**	**Model 3**	**Model 4**	**Model 5**	**Model 6**	**Model 7**	**Model 8**
Latino Treat.	-0.256^{***}	-0.270^{***}	-0.203^{***}	-0.211^{***}	-0.171^{***}	-0.182^{***}	-0.297^{***}	-0.306^{***}
	(0.046)	(0.045)	(0.043)	(0.043)	(0.041)	(0.041)	(0.063)	(0.062)
Immigrant Treat.	-0.266^{***}	-0.274^{***}	-0.145^{***}	-0.151^{***}	-0.179^{***}	-0.182^{***}	-0.235^{***}	-0.247^{***}
	(0.043)	(0.043)	(0.041)	(0.040)	(0.039)	(0.038)	(0.059)	(0.058)
Female		0.015		0.025		0.044		-0.062
		(0.037)		(0.035)		(0.033)		(0.051)
Education		-0.024^{**}		-0.007		-0.004		-0.009
		(0.011)		(0.010)		(0.010)		(0.015)
Income		0.012		0.009		0.006		-0.009
		(0.015)		(0.014)		(0.013)		(0.020)
Age		-0.0002		-0.001		0.0004		-0.003
		(0.001)		(0.001)		(0.001)		(0.002)
Mexican		-0.093^{**}		-0.066		-0.066^{*}		-0.047
		(0.044)		(0.042)		(0.039)		(0.060)
English L.		0.014		0.015		0.020		0.067
		(0.036)		(0.034)		(0.032)		(0.049)
Pol. Interest		0.023^{***}		0.019^{***}		0.019^{***}		0.029^{***}
		(0.006)		(0.006)		(0.006)		(0.009)
Constant	0.576^{***}	0.548^{***}	0.587^{***}	0.490^{***}	0.623^{***}	0.466^{***}	0.481^{***}	0.504^{***}
	(0.032)	(0.098)	(0.030)	(0.093)	(0.028)	(0.088)	(0.044)	(0.134)
N	324	324	324	324	324	324	324	324
R-squared	0.125	0.178	0.070	0.112	0.075	0.123	0.074	0.126
Adj. R-squared	0.120	0.155	0.064	0.086	0.069	0.098	0.069	0.101

$^{***}p < .01$; $^{**}p < .05$; $^{*}p < .1$

B.4 Candidate Evaluation ATEs: Asians

Table 14: Candidate Evaluation ATEs (Full Asian Sample)

	Cares<br>Model 1	Honest<br>Model 2	Hardworking<br>Model 3	Vote Stevens<br>Model 4
Asian Treatment	-0.254^{***}	-0.198^{***}	-0.179^{***}	-0.256^{***}
	(0.027)	(0.025)	(0.024)	(0.038)
Immigrant Treatment	-0.209^{***}	-0.147^{***}	-0.155^{***}	-0.192^{***}
	(0.027)	(0.025)	(0.024)	(0.038)
Constant	0.512^{***}	0.532^{***}	0.551^{***}	0.447^{***}
	(0.019)	(0.018)	(0.017)	(0.027)
N	815	815	815	815
R-squared	0.113	0.075	0.073	0.056
Adj. R-squared	0.110	0.073	0.071	0.054

$^{***}p < .01$; $^{**}p < .05$; $^{*}p < .1$

Table 15: Candidate Evaluation ATEs (Native Born Asians)

	Cares<br>Model 1	Cares<br>Model 2	Honest<br>Model 3	Honest<br>Model 4	Hardworking<br>Model 5	Hardworking<br>Model 6	Vote<br>Model 7	Vote<br>Model 8
Asian Treat.	-0.206^{***}	-0.206^{***}	-0.176^{***}	-0.176^{***}	-0.178^{***}	-0.180^{***}	-0.141^{**}	-0.146^{**}
	(0.048)	(0.048)	(0.046)	(0.047)	(0.044)	(0.045)	(0.066)	(0.066)
Immigrant Treat.	-0.190^{***}	-0.201^{***}	-0.136^{***}	-0.143^{***}	-0.191^{***}	-0.200^{***}	-0.133^{**}	-0.150^{**}
	(0.047)	(0.047)	(0.045)	(0.046)	(0.043)	(0.044)	(0.064)	(0.065)
Female		-0.023		-0.030		-0.035		-0.032
		(0.039)		(0.038)		(0.037)		(0.054)
Education		-0.011		-0.017		-0.006		-0.021
		(0.016)		(0.015)		(0.015)		(0.022)
Income		0.001		0.002		0.004		0.021
		(0.018)		(0.017)		(0.016)		(0.024)
Age		0.003		-0.0002		0.0004		0.002
		(0.002)		(0.002)		(0.002)		(0.003)
Indian		-0.008		-0.032		-0.033		-0.052
		(0.043)		(0.042)		(0.040)		(0.059)
English L.		-0.101		-0.110^{*}		-0.077		-0.202^{**}
		(0.066)		(0.064)		(0.061)		(0.090)
Pol. Interest		0.004		0.002		0.003		0.009
		(0.008)		(0.008)		(0.007)		(0.011)
Constant	0.441^{***}	0.495^{***}	0.500^{***}	0.689^{***}	0.530^{***}	0.622^{***}	0.316^{***}	0.457^{***}
	(0.034)	(0.117)	(0.033)	(0.113)	(0.031)	(0.109)	(0.047)	(0.160)
N	246	246	246	246	246	246	246	246
R-squared	0.088	0.106	0.062	0.088	0.089	0.104	0.024	0.060
Adj. R-squared	0.080	0.072	0.054	0.053	0.081	0.070	0.016	0.024

$^{***}p < .01$; $^{**}p < .05$; $^{*}p < .1$

Table 16: Candidate Evaluation ATEs (Foreign Born Asians)

	Cares<br>Model 1	Cares<br>Model 2	Honest<br>Model 3	Honest<br>Model 4	Hardworking<br>Model 5	Hardworking<br>Model 6	Vote<br>Model 7	Vote<br>Model 8
Asian Treat.	-0.274^{***}	-0.271^{***}	-0.207^{***}	-0.203^{***}	-0.180^{***}	-0.176^{***}	-0.306^{***}	-0.301^{***}
	(0.032)	(0.032)	(0.030)	(0.030)	(0.029)	(0.029)	(0.047)	(0.046)
Immigrant Treat.	-0.216^{***}	-0.207^{***}	-0.151^{***}	-0.143^{***}	-0.137^{***}	-0.130^{***}	-0.215^{***}	-0.208^{***}
	(0.032)	(0.032)	(0.030)	(0.030)	(0.029)	(0.029)	(0.047)	(0.047)
Female		0.018		0.042^{*}		0.043^{*}		0.036
		(0.027)		(0.025)		(0.024)		(0.039)
Education		0.007		0.007		0.008		0.012
		(0.010)		(0.010)		(0.009)		(0.015)
Income		-0.031^{**}		-0.025^{**}		-0.021^{*}		-0.054^{***}
		(0.012)		(0.012)		(0.011)		(0.018)
Age		0.001		-0.00000		0.001		0.001
		(0.001)		(0.001)		(0.001)		(0.001)
Indian		0.055^{**}		0.017		0.015		0.096^{**}
		(0.028)		(0.026)		(0.025)		(0.041)
English L.		-0.081^{***}		-0.053^{**}		-0.042^{*}		-0.051
		(0.028)		(0.027)		(0.026)		(0.041)
Pol. Interest		0.008		0.011^{**}		0.007		0.009
		(0.005)		(0.005)		(0.005)		(0.008)
Constant	0.543^{***}	0.524^{***}	0.545^{***}	0.526^{***}	0.559^{***}	0.520^{***}	0.503^{***}	0.482^{***}
	(0.023)	(0.076)	(0.022)	(0.072)	(0.021)	(0.070)	(0.033)	(0.112)
N	569	569	569	569	569	569	569	569
R-squared	0.126	0.157	0.081	0.106	0.069	0.089	0.074	0.102
Adj. R-squared	0.123	0.144	0.078	0.092	0.066	0.074	0.071	0.088

$^{***}p < .01$; $^{**}p < .05$; $^{*}p < .1$

C Part III – Supplementary Models

C.1 Emotion ATEs + Manipulation Check: Latinos

Table 17: Emotion ATEs (Latino Sample + Manipulation Check)

	**Sad**<br>**Model 1**	**Angry**<br>**Model 2**	**Afraid**<br>**Model 3**	**Enthusiastic**<br>**Model 4**	**Hopeful**<br>**Model 5**
Latino Treatment	0.248^{***}	0.393^{***}	0.108^{***}	-0.153^{***}	-0.218^{***}
	(0.026)	(0.025)	(0.027)	(0.023)	(0.024)
Immigrant Treatment	0.263^{***}	0.349^{***}	0.126^{***}	-0.132^{***}	-0.172^{***}
	(0.026)	(0.025)	(0.027)	(0.023)	(0.023)
Constant	0.415^{***}	0.342^{***}	0.287^{***}	0.339^{***}	0.418^{***}
	(0.019)	(0.018)	(0.019)	(0.016)	(0.017)
N	1096	1096	1096	1096	1096
R-squared	0.102	0.218	0.023	0.047	0.080
Adj. R-squared	0.101	0.216	0.022	0.045	0.078

$^{***}p < .01$; $^{**}p < .05$; $^{*}p < .1$

Table 18: Latino Emotion ATEs (By Nativity + Manipulation Check)

	**Sad**<br>**Model 1**	**Sad**<br>**Model 2**	**Angry**<br>**Model 3**	**Angry**<br>**Model 4**	**Afraid**<br>**Model 5**	**Afraid**<br>**Model 6**	**Enthus**<br>**Model 7**	**Enthus**<br>**Model 8**	**Hopeful**<br>**Model 9**	**Hopeful**<br>**Model 10**
Latino Treat.	0.250^{***}	0.249^{***}	0.400^{***}	0.391^{***}	0.098^{***}	0.130^{**}	-0.172^{***}	-0.128^{***}	-0.247^{***}	-0.184^{***}
	(0.030)	(0.056)	(0.027)	(0.054)	(0.030)	(0.058)	(0.025)	(0.049)	(0.026)	(0.048)
Immigrant Treat.	0.242^{***}	0.321^{***}	0.346^{***}	0.378^{***}	0.139^{***}	0.092^{*}	-0.126^{***}	-0.145^{***}	-0.166^{***}	-0.193^{***}
	(0.030)	(0.053)	(0.028)	(0.051)	(0.030)	(0.055)	(0.025)	(0.047)	(0.026)	(0.045)
Female	0.058^{**}	0.057	0.078^{***}	0.018	0.059^{**}	0.022	-0.036^{*}	-0.045	-0.029	0.036
	(0.025)	(0.045)	(0.023)	(0.043)	(0.025)	(0.047)	(0.021)	(0.040)	(0.022)	(0.039)
Education	0.010	-0.011	0.010	-0.004	0.014^{*}	0.004	-0.014^{*}	-0.006	-0.013	-0.017
	(0.009)	(0.014)	(0.008)	(0.013)	(0.009)	(0.015)	(0.007)	(0.012)	(0.008)	(0.012)
Income	-0.034^{***}	-0.006	-0.012	0.010	-0.016	-0.007	0.002	0.002	-0.0003	-0.002
	(0.011)	(0.018)	(0.010)	(0.018)	(0.011)	(0.019)	(0.009)	(0.016)	(0.010)	(0.015)
Age	0.001	0.001	0.001	0.0001	-0.002^{**}	-0.001	-0.003^{***}	-0.00004	-0.003^{***}	-0.002
	(0.001)	(0.001)	(0.001)	(0.001)	(0.001)	(0.001)	(0.001)	(0.001)	(0.001)	(0.001)
Mexican	-0.009	0.102^{*}	-0.006	0.138^{***}	-0.056^{**}	0.079	-0.020	-0.059	-0.023	-0.104^{**}
	(0.025)	(0.053)	(0.023)	(0.051)	(0.025)	(0.055)	(0.021)	(0.047)	(0.022)	(0.047)
English L.	-0.030	-0.061	-0.022	0.025	-0.065^{*}	-0.065	-0.093^{***}	-0.010	-0.086^{***}	-0.021
	(0.038)	(0.045)	(0.034)	(0.043)	(0.037)	(0.046)	(0.031)	(0.040)	(0.033)	(0.038)
Pol. Interest	0.009^{**}	0.015^{*}	0.006	0.006	0.015^{***}	0.019^{**}	0.011^{***}	0.020^{***}	0.008^{*}	0.021^{***}
	(0.005)	(0.008)	(0.004)	(0.008)	(0.005)	(0.008)	(0.004)	(0.007)	(0.004)	(0.007)
Constant	0.369^{***}	0.308^{**}	0.251^{***}	0.164	0.291^{***}	0.212^{*}	0.567^{***}	0.273^{**}	0.670^{***}	0.539^{***}
	(0.070)	(0.120)	(0.064)	(0.115)	(0.069)	(0.124)	(0.058)	(0.106)	(0.061)	(0.104)
N	824	272	824	272	824	272	824	272	824	324
R-squared	0.118	0.171	0.244	0.237	0.063	0.064	0.095	0.075	0.132	0.107
Adj. R-squared	0.108	0.142	0.236	0.210	0.053	0.031	0.085	0.043	0.122	0.081

$^{***}p < .01$; $^{**}p < .05$; $^{*}p < .1$

C.2 Emotion ATEs + Manipulation Check: Asians

Table 19: Emotion ATEs (Asian Sample + Manipulation Check)

	**Sad**	**Angry**	**Afraid**	**Enthusiastic**	**Hopeful**
	**Model 1**	**Model 2**	**Model 3**	**Model 4**	**Model 5**
South Asian Treatment	0.244^{***}	0.323^{***}	0.114^{***}	-0.226^{***}	-0.272^{***}
	(0.031)	(0.030)	(0.032)	(0.026)	(0.029)
Immigrant Treatment	0.180^{***}	0.289^{***}	0.104^{***}	-0.195^{***}	-0.224^{***}
	(0.031)	(0.030)	(0.032)	(0.026)	(0.029)
Constant	0.438^{***}	0.343^{***}	0.371^{***}	0.411^{***}	0.485^{***}
	(0.022)	(0.021)	(0.022)	(0.018)	(0.020)
N	697	697	697	697	697
R-squared	0.086	0.165	0.022	0.111	0.128
Adj. R-squared	0.084	0.163	0.020	0.108	0.126

$^{***}p < .01$; $^{**}p < .05$; $^{*}p < .1$

Table 20: Asian Emotion ATEs (By Nativity + Manipulation Check)

	**Sad**	**Sad**	**Angry**	**Angry**	**Afraid**	**Afraid**	**Enthus**	**Enthus**	**Hopeful**	**Hopeful**
	**Model 1**	**Model 2**	**Model 3**	**Model 4**	**Model 5**	**Model 6**	**Model 7**	**Model 8**	**Model 9**	**Model 10**
South Asian Treat.	0.222^{***}	0.261^{***}	0.371^{***}	0.313^{***}	0.085	0.140^{***}	-0.216^{***}	-0.227^{***}	-0.215^{***}	-0.298^{***}
	(0.057)	(0.038)	(0.050)	(0.037)	(0.056)	(0.038)	(0.039)	(0.033)	(0.047)	(0.035)
Immigrant Treat.	0.111^{*}	0.215^{***}	0.340^{***}	0.276^{***}	0.063	0.129^{***}	-0.138^{***}	-0.210^{***}	-0.196^{***}	-0.233^{***}
	(0.057)	(0.038)	(0.050)	(0.037)	(0.056)	(0.038)	(0.039)	(0.033)	(0.046)	(0.036)
Female	0.075	0.032	0.106^{**}	0.045	0.180^{***}	0.062^{*}	0.004	−0.025	−0.009	−0.023
	(0.047)	(0.032)	(0.041)	(0.031)	(0.046)	(0.032)	(0.032)	(0.028)	(0.038)	(0.030)
Education	0.031	0.018	0.020	0.003	0.053^{***}	0.035^{***}	−0.012	0.019^{*}	−0.004	−0.002
	(0.020)	(0.012)	(0.017)	(0.012)	(0.019)	(0.012)	(0.013)	(0.010)	(0.016)	(0.011)
Income	0.010	0.005	0.017	0.065^{***}	−0.009	−0.001	−0.001	-0.027^{**}	0.017	-0.043^{***}
	(0.021)	(0.015)	(0.019)	(0.014)	(0.021)	(0.015)	(0.015)	(0.013)	(0.017)	(0.014)
Age	0.001	0.001	−0.002	0.0001	−0.001	−0.001	−0.001	−0.0003	−0.001	0.001
	(0.002)	(0.001)	(0.002)	(0.001)	(0.002)	(0.001)	(0.002)	(0.001)	(0.002)	(0.001)
Indian	−0.005	0.001	−0.006	−0.028	0.030	−0.024	0.044	0.031	0.058	0.081^{**}
	(0.053)	(0.034)	(0.046)	(0.033)	(0.052)	(0.034)	(0.036)	(0.029)	(0.043)	(0.032)
English L.	−0.055	−0.004	−0.057	0.005	-0.154^{*}	0.006	−0.058	-0.088^{***}	−0.073	-0.080^{**}
	(0.086)	(0.034)	(0.075)	(0.033)	(0.085)	(0.034)	(0.059)	(0.030)	(0.070)	(0.032)
Pol. Interest	−0.004	0.009	−0.002	0.010	0.006	0.003	0.005	0.003	0.001	−0.002
	(0.009)	(0.006)	(0.008)	(0.006)	(0.009)	(0.006)	(0.006)	(0.006)	(0.007)	(0.006)
Constant	0.303^{**}	0.225^{**}	0.321^{***}	0.001	0.257^{*}	0.163^{*}	0.364^{***}	0.510^{***}	0.399^{***}	0.678^{***}
	(0.140)	(0.093)	(0.123)	(0.090)	(0.138)	(0.092)	(0.096)	(0.080)	(0.114)	(0.086)
N	210	487	210	487	210	487	210	487	210	487
R-squared	0.096	0.110	0.279	0.188	0.117	0.055	0.155	0.144	0.139	0.174
Adj. R-squared	0.055	0.093	0.247	0.173	0.077	0.037	0.117	0.128	0.100	0.158

$^{***}p < .01$; $^{**}p < .05$; $^{*}p < .1$

C.3 Candidate Evaluation ATEs + Manipulation Check: Latinos

Table 21: Candidate Evaluation ATEs (Latino Sample + Manipulation Check)

	**Cares**<br>**Model 1**	**Honest**<br>**Model 2**	**Hardworking**<br>**Model 3**	**Vote Stevens**<br>**Model 4**
Latino Treatment	-0.290^{***}	-0.233^{***}	-0.215^{***}	-0.242^{***}
	(0.022)	(0.022)	(0.021)	(0.031)
Immigrant Treatment	-0.250^{***}	-0.159^{***}	-0.167^{***}	-0.161^{***}
	(0.022)	(0.022)	(0.021)	(0.031)
Constant	0.512^{***}	0.530^{***}	0.568^{***}	0.389^{***}
	(0.016)	(0.016)	(0.015)	(0.022)
N	1096	1096	1096	1096
R-squared	0.157	0.098	0.094	0.053
Adj. R-squared	0.156	0.096	0.092	0.051

$^{***}p < .01$; $^{**}p < .05$; $^{*}p < .1$

Table 22: Latino Candidate Evaluation ATEs (By Nativity + Manipulation Check)

	**Cares**<br>**Native**<br>**Model 1**	**Cares**<br>**Foreign**<br>**Model 2**	**Honest**<br>**Native**<br>**Model 3**	**Honest**<br>**Foreign**<br>**Model 4**	**Hardworking**<br>**Native**<br>**Model 5**	**Hardworking**<br>**Foreign**<br>**Model 6**	**Vote**<br>**Native**<br>**Model 7**	**Vote**<br>**Foreign**<br>**Model 8**
Latino Treat.	-0.308^{***}	-0.278^{***}	-0.249^{***}	-0.207^{***}	-0.233^{***}	-0.186^{***}	-0.241^{***}	-0.285^{***}
	(0.024)	(0.049)	(0.024)	(0.048)	(0.024)	(0.045)	(0.035)	(0.066)
Immigrant Treat.	-0.243^{***}	-0.294^{***}	-0.167^{***}	-0.155^{***}	-0.162^{***}	-0.204^{***}	-0.140^{***}	-0.251^{***}
	(0.025)	(0.047)	(0.025)	(0.045)	(0.024)	(0.043)	(0.036)	(0.063)
Female	-0.057^{***}	0.021	-0.010	0.016	-0.029	0.045	-0.050^{*}	-0.064
	(0.020)	(0.039)	(0.021)	(0.038)	(0.020)	(0.036)	(0.030)	(0.053)
Education	-0.008	-0.028^{**}	-0.006	-0.003	-0.005	-0.003	-0.027^{**}	-0.020
	(0.007)	(0.012)	(0.007)	(0.012)	(0.007)	(0.011)	(0.010)	(0.017)
Income	0.006	0.033^{**}	0.008	0.020	0.008	0.019	0.021	0.020
	(0.009)	(0.016)	(0.009)	(0.016)	(0.009)	(0.015)	(0.013)	(0.022)
Age	-0.001^{*}	-0.0001	-0.002^{**}	-0.001	-0.0002	0.0002	-0.003^{***}	-0.003
	(0.001)	(0.001)	(0.001)	(0.001)	(0.001)	(0.001)	(0.001)	(0.002)
Mexican	-0.023	-0.058	-0.042^{**}	-0.037	-0.038^{*}	-0.041	-0.080^{***}	0.010
	(0.020)	(0.046)	(0.021)	(0.045)	(0.020)	(0.042)	(0.030)	(0.063)
English L.	-0.062^{**}	0.036	-0.032	0.044	-0.004	0.040	-0.033	0.057
	(0.031)	(0.039)	(0.031)	(0.038)	(0.030)	(0.036)	(0.044)	(0.053)
Pol. Interest	0.002	0.017^{**}	0.007^{*}	0.012^{*}	0.007^{**}	0.012^{*}	0.006	0.028^{***}
	(0.004)	(0.007)	(0.004)	(0.007)	(0.004)	(0.006)	(0.006)	(0.009)
Constant	0.663^{***}	0.489^{***}	0.615^{***}	0.447^{***}	0.557^{***}	0.454^{***}	0.650^{***}	0.445^{***}
	(0.057)	(0.105)	(0.057)	(0.102)	(0.056)	(0.096)	(0.082)	(0.142)
N	824	272	824	272	824	272	824	272
R-squared	0.189	0.183	0.127	0.095	0.119	0.117	0.079	0.128
Adj. R-squared	0.180	0.155	0.118	0.064	0.109	0.086	0.069	0.098

$^{***}p < .01$; $^{**}p < .05$; $^{*}p < .1$

C.4 Candidate Evaluation ATEs + Manipulation Check: Asians

Table 23: Candidate Evaluation ATEs (Asian Sample + Manipulation Check)

	Cares<br>Model 1	Honest<br>Model 2	Hardworking<br>Model 3	Vote Stevens<br>Model 4
Asian Treatment	-0.310^{***}	-0.239^{***}	-0.220^{***}	-0.320^{***}
	(0.027)	(0.026)	(0.025)	(0.039)
Immigrant Treatment	-0.275^{***}	-0.186^{***}	-0.200^{***}	-0.256^{***}
	(0.027)	(0.026)	(0.025)	(0.039)
Constant	0.515^{***}	0.529^{***}	0.553^{***}	0.454^{***}
	(0.019)	(0.018)	(0.018)	(0.027)
N	697	697	697	697
R-squared	0.190	0.120	0.120	0.100
Adj. R-squared	0.188	0.117	0.118	0.097

$^{***}p < .01$; $^{**}p < .05$; $^{*}p < .1$

Table 24: Asian Candidate Evaluation ATEs (By Nativity + Manipulation Check)

	Cares<br>Native<br>Model 1	Cares<br>Foreign<br>Model 2	Honest<br>Native<br>Model 3	Honest<br>Foreign<br>Model 4	Hardworking<br>Native<br>Model 5	Hardworking<br>Foreign<br>Model 6	Vote<br>Native<br>Model 7	Vote<br>Foreign<br>Model 8
Asian Treat.	-0.251^{***}	-0.334^{***}	-0.211^{***}	-0.249^{***}	-0.206^{***}	-0.223^{***}	-0.188^{***}	-0.376^{***}
	(0.047)	(0.032)	(0.049)	(0.031)	(0.046)	(0.030)	(0.066)	(0.047)
Immigrant Treat.	-0.267^{***}	-0.276^{***}	-0.168^{***}	-0.189^{***}	-0.247^{***}	-0.178^{***}	-0.183^{***}	-0.289^{***}
	(0.046)	(0.032)	(0.048)	(0.031)	(0.046)	(0.030)	(0.065)	(0.047)
Female	0.008	-0.007	0.0002	0.019	-0.021	0.023	0.043	0.001
	(0.038)	(0.027)	(0.040)	(0.026)	(0.038)	(0.026)	(0.054)	(0.040)
Education	-0.001	0.004	-0.015	0.007	0.002	0.008	-0.016	0.005
	(0.016)	(0.010)	(0.017)	(0.010)	(0.016)	(0.010)	(0.023)	(0.015)
Income	0.012	-0.033^{***}	0.012	-0.026^{**}	0.009	-0.025^{**}	0.012	-0.069^{***}
	(0.017)	(0.013)	(0.018)	(0.012)	(0.017)	(0.012)	(0.025)	(0.018)
Age	0.002	0.001	-0.001	0.0001	-0.0004	0.001	0.001	0.002
	(0.002)	(0.001)	(0.002)	(0.001)	(0.002)	(0.001)	(0.003)	(0.001)
Indian	0.008	0.083^{***}	-0.032	0.036	-0.013	0.031	-0.004	0.125^{***}
	(0.043)	(0.028)	(0.045)	(0.028)	(0.043)	(0.027)	(0.061)	(0.042)
English L.	-0.081	-0.073^{**}	-0.080	-0.040	-0.076	-0.023	-0.179^{*}	-0.045
	(0.070)	(0.029)	(0.073)	(0.028)	(0.069)	(0.027)	(0.099)	(0.042)
Pol. Interest	-0.004	0.0001	-0.005	0.006	-0.003	0.003	-0.001	0.002
	(0.007)	(0.005)	(0.008)	(0.005)	(0.007)	(0.005)	(0.010)	(0.008)
Constant	0.427^{***}	0.590^{***}	0.651^{***}	0.554^{***}	0.602^{***}	0.549^{***}	0.461^{***}	0.602^{***}
	(0.115)	(0.078)	(0.119)	(0.076)	(0.113)	(0.073)	(0.161)	(0.114)
N	210	487	210	487	210	487	210	487
R-squared	0.184	0.239	0.116	0.147	0.152	0.131	0.074	0.164
Adj. R-squared	0.148	0.224	0.076	0.131	0.114	0.115	0.032	0.148

$^{***}p < .01$; $^{**}p < .05$; $^{*}p < .1$

C.5 Immigrant Identity

Figure A1: **Immigrant Identity by Immigrant Status and Treatment Condition**

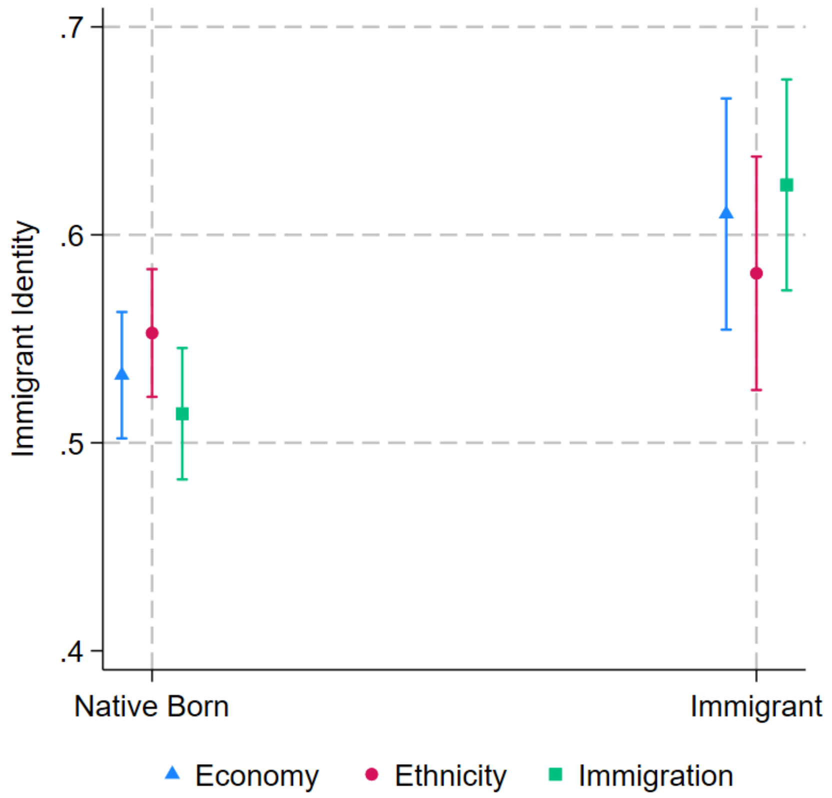

Note: Mean values of each group on a four item IDPG Immigrant Identity Scale
